# Supplementary material for: Molecular motor-driven reversible liquid-liquid phase separation of supramolecular assemblies
Source: Nat Commun. 2025 Nov 14;16:10017. doi: 10.1038/s41467-025-64993-9 (PMC12618938; doi:10.1038/s41467-025-64993-9)
Supplement: Supplementary file 1 — Supplementary Information [file 41467_2025_64993_MOESM1_ESM.pdf]

# Supplementary Information

# Molecular Motor-driven Reversible Liquid-liquid Phase Separation of Supramolecular Assemblies

Fan Xu,[1] Marco Ovalle,[1] Youxin Fu,[1] Marc A. C. Stuart,[2] and Ben L. Feringa[1]*

[1]Synthetic Organic Chemistry, Stratingh Institute for Chemistry, University of Groningen, Nijenborgh 3, 9747 AG Groningen, The Netherlands.

[2]Groningen Biomolecular Sciences and Biotechnology Institute, University of Groningen, Nijenborgh 7, 9747 AG Groningen, The Netherlands.

Present address:

Fan Xu: Institute for Complex Molecular Systems and Laboratory of Macromolecular and Organic Chemistry, Eindhoven University of Technology, 5600 MB, Eindhoven, The Netherlands.

Marco Ovalle: IMDEA Nanociencia, c/Faraday 9, Madrid 28049, Spain.

Youxin Fu: College of Science, Nanjing Forestry University, Nanjing, 210037, China.

*Correspondence: b.l.feringa@rug.nl

## Table of Contents


Supplementary Methods ........ 3
1. General information ........ 3
2. Synthesis ........ 4
Supplementary Discussion ........ 6
1. Ultraviolet–visible (UV-Vis) spectroscopy study ........ 6
2. Transmittance measurement ........ 9
3. Cryogenic transmission electron microscopy (Cryo-TEM) study ........ 10
4. Dynamic light scattering (DLS) measurements ........ 10
5. Fourier transform infrared (FTIR) spectroscopy study ........ 13
6. Nile Red fluorescence assay ........ 13
7. Confocal laser scanning microscopy (CLSM) study ........ 14
8. Fluorescence recovery after photobleaching (FRAP) ........ 15
9. Eyring analysis on the thermal helix inversion of molecular motors ........ 15
10. Density functional theory (DFT) calculations ........ 20
Supplementary Notes ........ 21
Supplementary Figures ........ 22
1. NMR data ........ 22
2. HRMS data ........ 28
Supplementary References ........ 29

# Supplementary Methods

## 1. General information

All commercially available chemicals were purchased from Acros, Aldrich, or TCI, and were used as received. Solvents used in the reactions were dried using an MBraun SPS-800 solvent purification system or purchased from Acros. The water (ULC/MS grade) used in sample preparation was purchased from Biosolve. Analytical TLC was carried out on Merck silica gel 60 F254 plates, and visualization was accomplished under UV light. Column chromatography was performed on a Reveleris X2 Flash Chromatography system. NMR spectra were recorded on Varian AMX400 ($^1$H: 400 MHz, $^{13}$C: 101 MHz) and Varian Unity Plus ($^1$H: 500 MHz, $^{13}$C: 125 MHz) spectrometers. Chemical shifts ($\delta$) are expressed relative to the resonances of the residual non-deuterated solvent for $^1$H NMR [$CDCl_3$: $^1$H(δ) = 7.26 ppm] and $^{13}$C NMR [$CDCl_3$: $^{13}$C(δ) = 77.2 ppm]. Absolute values of the coupling constants are given in Hertz (Hz), regardless of their sign. Multiplicities are abbreviated as singlet (s), doublet (d), doublet of doublets (dd), triplet (t), triplet of doublets (td), quartet (q), multiplet (m), and broad (br). High-resolution mass spectrometry (HRMS) was performed on an LTQ Orbitrap XL spectrometer with ESI ionization.

## 2. Synthesis

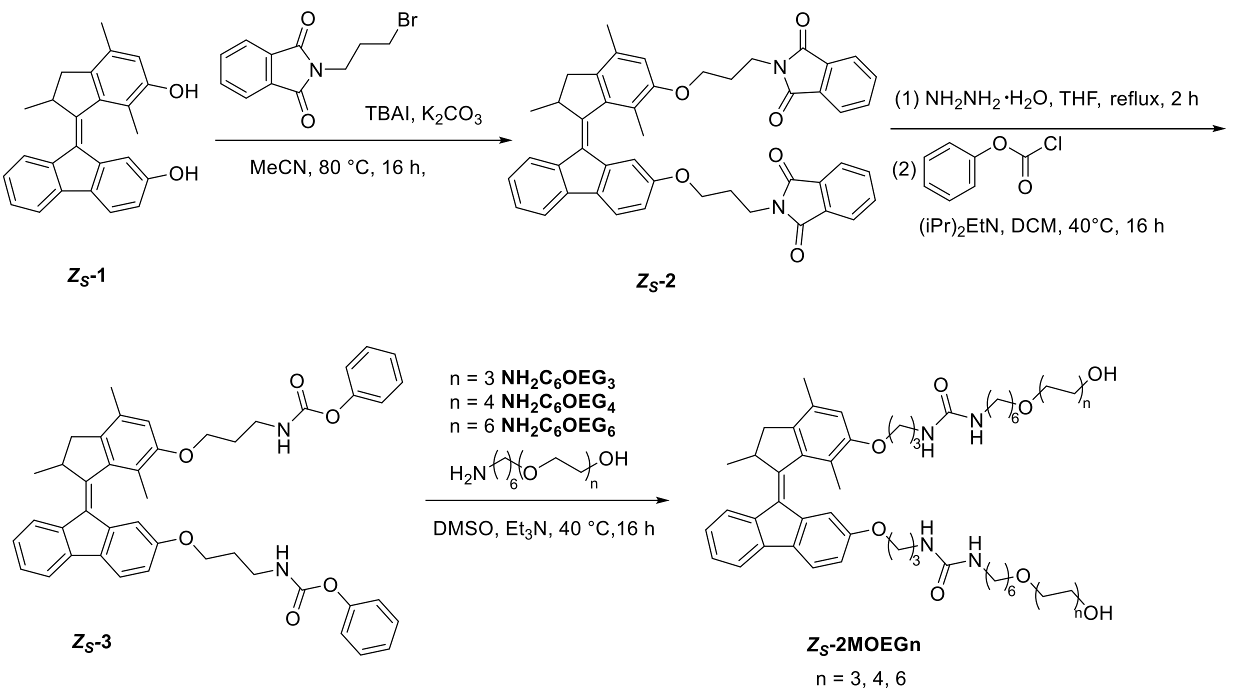


**Supplementary Figure 1.** Scheme of the synthesis of ***Z*** $_S$**-2MOEGn**.

The precursor ***Z*** $_S$**-1** was synthesized from hydroxy-substituted fluoren-9-ylidene hydrazine and trimethyl indanone through a Barton–Kellogg reaction. The detailed procedure has been reported in the authors' previous publication.[1] The precursors **$NH_2C_6OEG_3$**, **$NH_2C_6OEG_4$**, and **$NH_2C_6OEG_6$** were synthesized from *N*-(6-bromohexyl)phthalimide and oligo(ethylene glycol) monomethyl ether via Williamson etherification, followed by deprotection of the amine using hydrazine. The full experimental details are available in the authors' earlier works.[2,3]

### *Z*$_S$-2

To a suspension of ***Z*** $_S$**-1** (284 mg, 0.8 mmol) in MeCN (8 mL) was added 2-(3-bromopropyl) isoindoline-1,3-dione (644 mg, 2.4 mmol), tetrabutylammonium iodide (890 mg, 2.4 mmol) and $K_2CO_3$ (445 g, 3.2 mmol) under a nitrogen atmosphere. The mixture was stirred at 80 °C overnight followed by concentrating *in vacuo*. To the concentrated mixture was added 15 mL deionized water. The aqueous phase was extracted with EtOAc. The organic layer was washed with brine, dried over $Na_2SO_4$, and concentrated *in vacuo*. The crude product was purified by column chromatography ($SiO_2$, EtOAc:pentane = 1:3) to afford compound ***Z*** $_S$**-2** (357 mg, 61%) as a yellow solid. $^1$H NMR (400 MHz, $CDCl_3$) δ 7.87 – 7.71 (m, 5H), 7.69 – 7.60 (m, 5H), 7.55 (d, *J* = 8.3 Hz, 1H), 7.28 (dt, *J* = 18.9, 6.8 Hz, 2H), 6.94 (d, *J* = 2.4 Hz, 1H), 6.73 (dd, *J* = 8.3, 2.4 Hz, 1H), 6.70 (s, 1H), 4.15 (d, *J* = 5.7 Hz, 2H), 4.04 (dt, *J* = 9.4, 6.0 Hz, 1H), 3.92 (q, *J* = 7.1 Hz, 3H), 3.84 (q, *J* = 7.0 Hz, 3H), 3.14 (dd, *J* = 14.5, 5.8 Hz, 1H), 2.51 (d, *J* = 14.5 Hz, 1H), 2.29 (s, 3H), 2.22 (q, *J* = 6.5 Hz, 2H), 2.11 (s, 5H), 1.30 (d, *J* = 6.8 Hz, 3H). $^{13}$C NMR (126 MHz, cdcl3) δ 168.5, 168.4, 158.5, 156.6, 152.6, 141.0, 140.2, 139.7, 139.4, 137.1, 134.0, 133.9, 133.0, 132.3, 132.1, 130.6, 127.0, 125.8, 124.0, 123.8, 123.3, 123.3, 119.8, 119.0, 114.9, 113.8, 109.4, 66.3, 65.6, 44.2, 39.4, 35.7, 35.6, 29.1, 28.5, 19.3, 19.0, 15.8. HRMS (ESI+) calcd. for $[M+H]^+$: 730.2993, found: 730.2975.

### *Z*$_S$-3

To a suspension of ***Z*** $_S$**-2** (350 mg, 0.48 mmol) in EtOH (10 mL) was added hydrazine hydrate (50–60%, 7.2 mmol, 0.56 mL), followed by heating the mixture at reflux for 2 h. After cooling and concentrating *in*

*vacuo*, the mixture was dissolved in 15% aq. NaOH (10 mL), and extracted with DCM. The organic layer was washed with brine, dried over $Na_2SO_4$, and then concentrated *in vacuo* to afford the compound (209 mg) as a yellow solid. The resulting compound (200 mg, 0.43 mmol) and *N*,*N*-diisopropylethylamine (200 µL, 0.94 mmol) were added to a solution of phenyl chloroformate (118 µL, 0.94 mmol) in dry DCM (3 mL) After stirring for 16 h at room temperature, the reaction mixture was concentrated *in vacuo.* The crude product was purified by column chromatography ($SiO_2$, EtOAc:pentane = 3:7) to afford compound ***Z<sub>S</sub>*-3** (177 mg, 54 %) as a yellow solid. $^1$H NMR (400 MHz, $CDCl_3$) δ 7.84 (d, *J* = 7.5 Hz, 1H), 7.70 (d, *J* = 7.5 Hz, 1H), 7.61 (d, *J* = 8.3 Hz, 1H), 7.38 – 7.28 (m, 6H), 7.19 (d, *J* = 6.9 Hz, 2H), 7.13 – 7.05 (m, 4H), 7.02 (d, *J* = 2.3 Hz, 1H), 6.85 (dd, *J* = 8.3, 2.3 Hz, 1H), 6.72 (s, 1H), 5.62 (s, 1H), 5.42 (s, 1H), 4.18 – 4.08 (m, 3H), 3.97 – 3.85 (m, 2H), 3.49 (q, *J* = 6.5 Hz, 2H), 3.44 – 3.35 (m, 2H), 3.16 (dd, *J* = 14.7, 5.7 Hz, 1H), 2.54 (d, *J* = 14.6 Hz, 1H), 2.30 (s, 3H), 2.20 (s, 3H), 2.16 – 2.06 (m, 2H), 1.98 (q, *J* = 6.3 Hz, 2H), 1.33 (d, *J* = 6.7 Hz, 3H).$^{13}$C NMR (101 MHz, $CDCl_3$) δ 158.4, 156.5, 155.0, 152.5, 151.2, 141.2, 140.2, 139.7, 139.5, 137.4, 133.3, 132.4, 130.7, 129.4, 129.4, 127.2, 126.0, 125.4, 125.4, 124.1, 123.4, 121.8, 121.7, 120.0, 119.1, 114.8, 113.9, 109.7, 67.1, 66.0, 44.2, 39.5, 39.3, 39.1, 29.8, 29.4, 19.3, 19.0, 16.1. HRMS (ESI+) calcd. for $[M+H]^+$: 710.3306, found: 710.3290

***Z<sub>S</sub>*-2MOEG6**

Triethylamine (25 µL, 178 µmol) and compound **$NH_2C_6OEG_6$** (68 mg, 178 µmol) were added to a solution of ***Z<sub>S</sub>*-3** (50.0 mg, 71 µmol) in dry DMSO (1.5 mL) under a nitrogen atmosphere. The mixture was stirred at 60 ºC for 16 h. After cooling to room temperature, water (3 mL) was added to the solution, followed by extraction with EtOAc. The organic layer was dried with $Na_2SO_4$ and concentrated *in vacuo*. The resulting viscous oil was dissolved in DCM (1.5 mL) and the product precipitated by adding pentane (6 mL). The precipitation was repeated three times. After drying *in vacuo*, ***Z<sub>S</sub>*-2MOEG6** (62.0 mg, 68%) was obtained as a yellow sticky solid. $^1$H NMR (400 MHz, $CDCl_3$) δ 7.82 (d, *J* = 7.4 Hz, 1H), 7.67 (d, *J* = 7.2 Hz, 1H), 7.57 (d, *J* = 8.3 Hz, 1H), 7.35 – 7.26 (m, 2H), 7.00 (d, *J* = 2.3 Hz, 1H), 6.81 (dd, *J* = 8.4, 2.3 Hz, 1H), 6.72 (s, 1H), 5.79 (s, 1H), 5.49 – 5.38 (m, 2H), 5.21 (s, 1H), 4.15 – 4.01 (m, 3H), 3.84 (t, *J* = 6.2 Hz, 2H), 3.70 (t, *J* = 4.4 Hz, 4H), 3.64 – 3.53 (m, 44H), 3.49 – 3.37 (m, 6H), 3.32 – 3.20 (m, 3H), 3.18 – 3.05 (m, 6H), 2.52 (d, *J* = 14.6 Hz, 1H), 2.28 (s, 3H), 2.13 (s, 3H), 2.06 – 1.94 (m, 2H), 1.94 – 1.80 (m, 2H), 1.58 – 1.50 (m, 4H), 1.47 – 1.40 (m, 4H), 1.34 – 1.28 (m, 11H). $^{13}$C NMR (151 MHz, $CDCl_3$) δ 159.5, 159.3, 156.7, 152.7, 140.1, 139.6, 139.4, 132.3, 130.5, 127.1, 126.0, 124.1, 123.4, 119.9, 119.0, 115.2, 113.8, 109.3, 77.4, 77.2, 77.0, 72.7, 72.7, 71.5, 71.3, 70.8, 70.7, 70.7, 70.7, 70.6, 70.4, 70.4, 70.2, 66.4, 65.6, 61.8, 49.2, 44.0, 40.4, 40.2, 39.4, 37.6, 37.2, 30.7, 30.5, 30.2, 29.6, 29.5, 26.9, 26.7, 26.0, 26.0, 19.3, 18.9, 16.1. HRMS (ESI+) calcd. for $[M+H]^+$: 1281.7742, found: 1281.7717.

***Z<sub>S</sub>*-2MOEG4**

Triethylamine (25 µL, 178 µmol) and compound **$NH_2C_6OEG_4$** (52 mg, 178 µmol), which was synthesized as described in our previous paperwas added to a solution of ***Z<sub>S</sub>*-3** (50.0 mg, 71 µmol) in DMSO (1.5 mL) under a nitrogen atmosphere. The mixture was stirred at 60 ºC for 16 h. After cooling to room temperature, water (3 mL) was added to the solution, followed by extraction with EtOAc. The organic layer was dried with $Na_2SO_4$ and concentrated *in vacuo*. The resulting viscous oil was dissolved in DCM (1.5 mL) and the product precipitated by adding pentane (6 mL). The precipitation was repeated three times. After drying *in vacuo*, ***Z<sub>S</sub>*-2MOEG4** (64.0 mg, 59 µmol, 81%) was obtained as a yellow sticky solid. $^1$H NMR (400 MHz, $CDCl_3$) δ 7.82 (d, *J* = 7.3 Hz, 1H), 7.67 (dd, *J* = 7.2, 1.7 Hz, 1H), 7.58 (d, *J* = 8.3 Hz, 1H), 7.35 – 7.26 (m, 2H), 7.00 (d, *J* = 2.3 Hz, 1H), 6.81 (dd, *J* = 8.3, 2.4 Hz, 1H), 6.73 (s, 1H), 5.80 (s, 1H), 5.50 – 5.41 (m, 2H), 5.32 (s, 1H), 4.15 – 4.03 (m, 3H), 3.84 (t, *J* = 6.2 Hz, 2H), 3.72 – 3.68 (m, 4H), 3.64 – 3.54 (m, 28H), 3.43 (q, *J* = 6.5 Hz,

5H), 3.34 – 3.22 (m, 3H), 3.21 – 3.03 (m, 6H), 2.53 (d, $J$ = 14.5 Hz, 1H), 2.29 (s, 3H), 2.14 (s, 3H), 2.04 – 1.95 (m, 2H), 1.93 – 1.81 (m,2H), 1.58 – 1.52 (m, 4H), 1.50 – 1.40 (dt, $J$ = 9.1, 6.9 Hz, 4H), 1.35 – 1.29 (m, 11H). $^{13}$C NMR (101 MHz, $CDCl_3$) δ 159.5, 159.4, 158.4, 156.7, 152.7, 141.0, 140.2, 139.6, 139.4, 136.7, 133.1, 132.3, 130.5, 127.1, 125.9, 124.1, 123.4, 119.9, 119.0, 115.2, 113.8, 109.2, 72.9, 72.8, 72.8, 71.5, 71.4, 70.8, 70.7, 70.7, 70.6, 70.6, 70.4, 70.3, 70.2, 70.2, 66.4, 65.6, 61.8, 61.7, 44.1, 40.3, 40.2, 39.4, 37.5, 37.1, 30.8, 30.4, 30.2, 30.0, 29.6, 29.5, 26.9, 26.7, 26.0, 25.9, 19.3, 18.9, 16.0. HRMS (ESI+) calcd. for $[M+H]^+$: 1107.6839, found: 1107.6818.

***$Z_S$*-2MOEG3**

Triethylamine (43.2 µL, 0.31 mmol) and compound **$NH_2C_6OEG_3$** (44 mg, 176 µmol), which was synthesized as described in our previous paper. [4] was added to a solution of ***$Z_S$*-3** (50.0 mg, 71 µmol) in DMSO (1.5 mL) under a nitrogen atmosphere. The mixture was stirred at 60 ºC for 16 h. After cooling to room temperature, water (3 mL) was added to the solution, followed by extraction with EtOAc. The organic layer was dried with $Na_2SO_4$ and concentrated *in vacuo*. The resulting viscous oil was dissolved in DCM (1.5 mL) and the product precipitated by adding pentane (6 mL). The precipitation was repeated three times. After drying *in vacuo*, pure ***$Z_S$*-2MOEG3** (43.0 mg, 59%) was obtained as a yellow sticky solid. $^1$H NMR (400 MHz, $CDCl_3$) 7.82 (d, $J$ = 7.3 Hz, 1H), 7.68 (d, $J$ = 7.2 Hz, 1H), 7.58 (d, $J$ = 8.2 Hz, 1H), 7.35 – 7.23 (m,2H), 7.00 (s, 1H), 6.82 (d, $J$ = 8.3 Hz, 1H), 6.72 (s, 1H), 5.71 (s, 1H), 5.33 (d, $J$ = 19.9 Hz, 2H), 5.11 (s, 1H), 4.15 – 4.02 (m, 3H), 3.84 (t, $J$ = 6.2 Hz, 2H), 3.71 (s, 4H), 3.66 – 3.54 (m, 20H), 3.42 (q, $J$ = 6.5 Hz, 5H), 3.34 – 3.24 (m, 3H), 3.18 – 3.05 (m, 6H), 2.53 (d, $J$ = 14.6 Hz, 1H), 2.29 (s, 3H), 2.14 (s, 3H), 2.05 – 1.94 (m, 2H), 1.94 – 1.82 (m, 2H), 1.5 – 1.51 (m, 4H), 1.49 – 1.42 (m, 4H), 1.36 – 1.28 (m, 11H).$^{13}$C NMR (151 MHz, $CDCl_3$) δ 159.5, 159.4, 158.4, 156.6, 152.7, 141.0, 140.1, 139.6, 139.4, 136.7, 133.1, 132.3, 130.5, 127.1, 126.0, 124.1, 123.4, 119.9, 119.0, 115.1, 113.8, 109.3, 72.7, 72.7, 71.5, 71.4, 70.7, 70.7, 70.4, 70.4, 70.1, 70.1, 66.4, 65.6, 61.7, 61.7, 44.1, 40.3, 40.2, 39.4, 37.6, 37.2, 30.7, 30.4, 30.2, 30.1, 29.6, 29.5, 26.8, 26.7, 25.9, 25.9, 19.3, 18.9, 16.0. HRMS (ESI+) calcd. for $[M+H]^+$: 1020.6349, found: 1020.6356.

## Supplementary Discussion

### 1. Ultraviolet–visible (UV-Vis) spectroscopy study

UV-Vis spectra were recorded on a Hewlett-Packard HP 8543 spectrometer in a quartz cuvette with a 1 cm path length. Irradiation of samples was carried out *in situ* using an LED light M365FP1 (5.29 mW watt model) positioned at a distance of 2 cm from the sample.

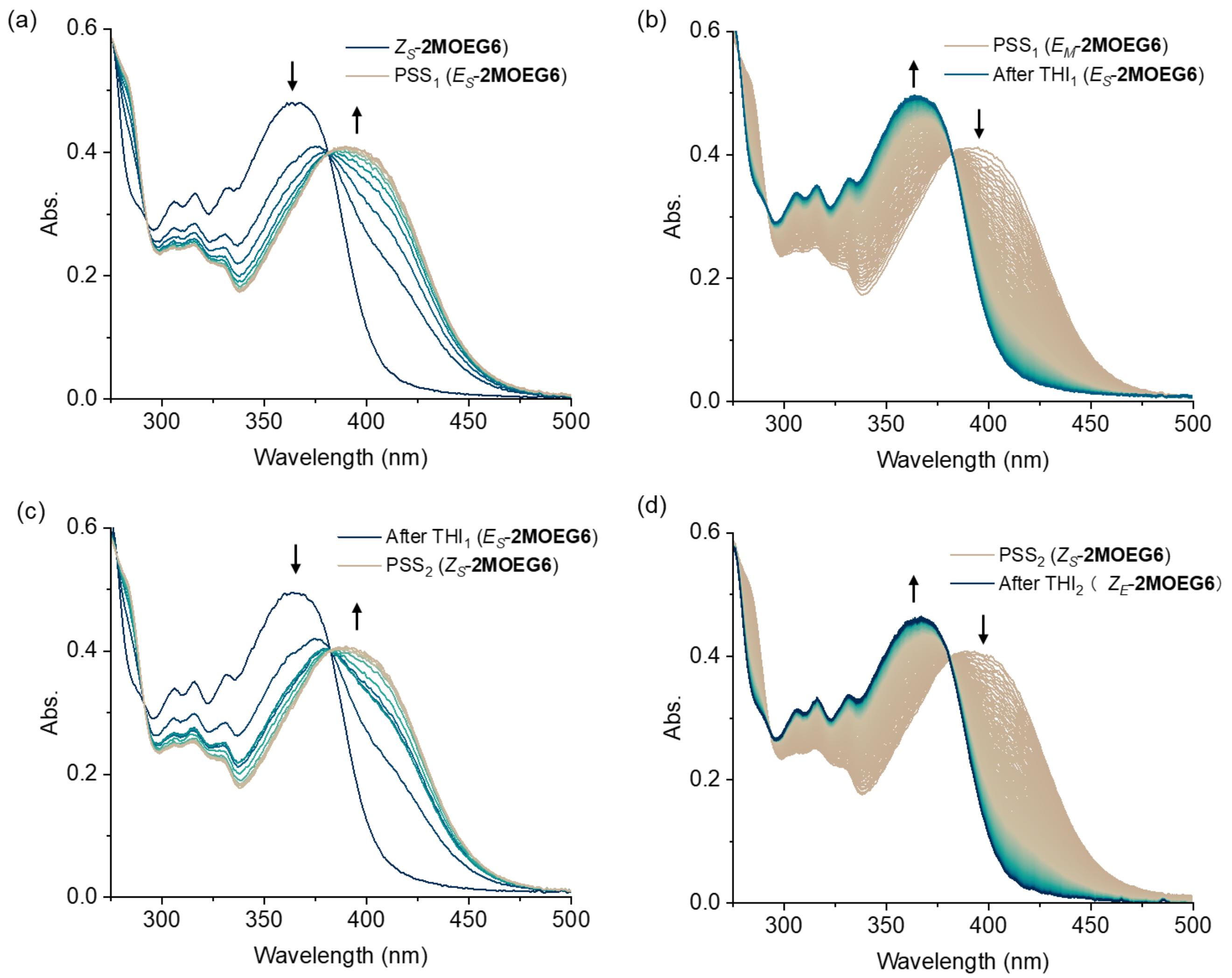


**Supplementary Figure 2. UV-Vis spectroscopy study of the rotation of $Z_S$-2MOEG6 in MeOH.** UV-Vis absorption spectra of **$Z_S$-2MOEG6** (50 µM, −3 ºC) in MeOH (a) upon 365 nm light irradiation for 3 min to get **$E_M$-2MOEG6**, (b) keeping in the dark for 3.5 h to reach **$E_S$-2MOEG6**, (c) subsequent irradiating with 365 nm light for 3 min to get **$Z_M$-2MOEG6**, (d) finally keeping in the dark for 4.5 h to recover **$Z_S$-2MOEG6**.

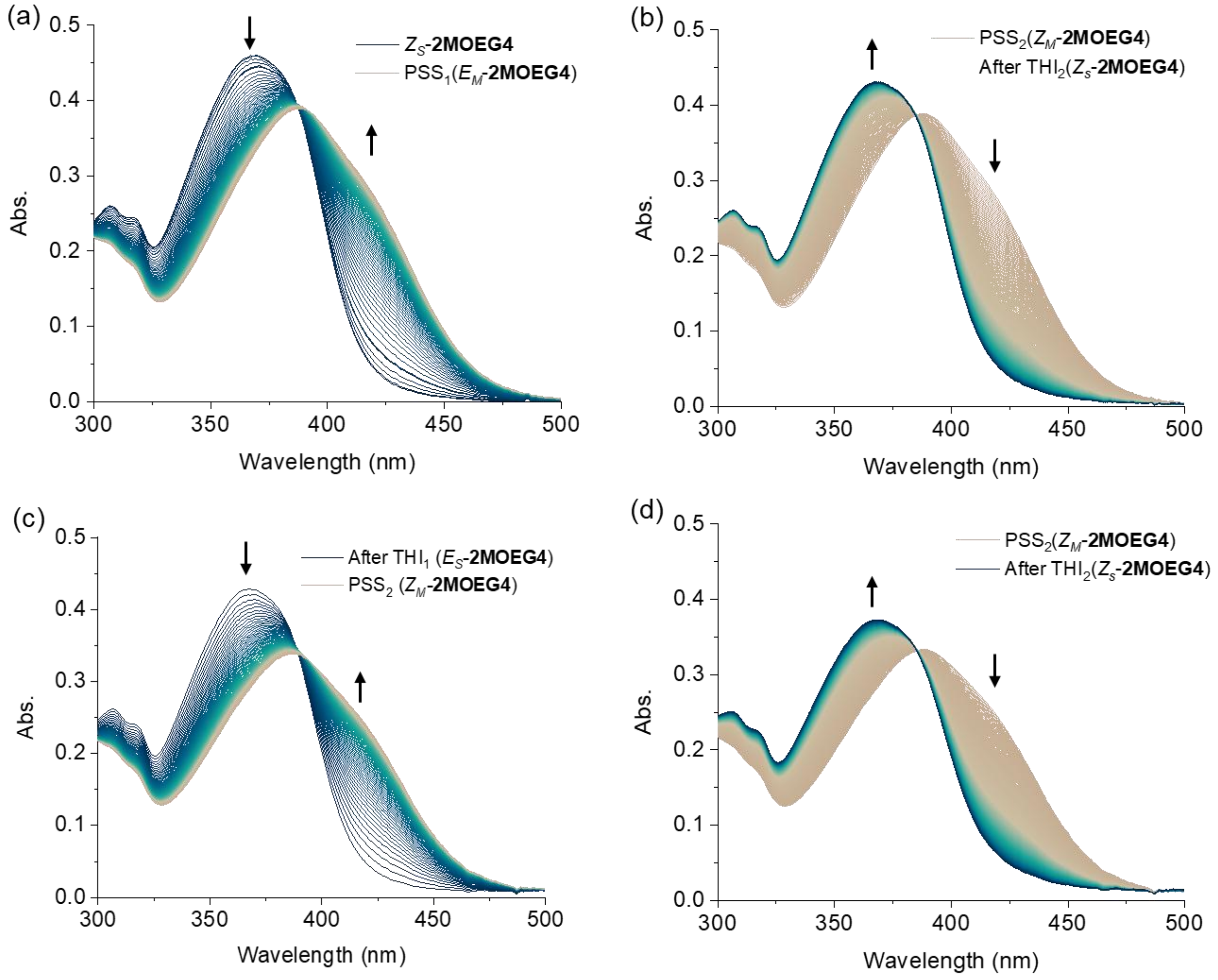


**Supplementary Figure 3. UV-Vis spectroscopy study of the rotation of *$Z_S$*-2MOEG4 in MeOH.** UV-Vis absorption spectra of ***$Z_S$*-2MOEG4** (50 µM, 5 ºC) in water (a) upon 365 nm light irradiation for 10 min to get ***$E_M$*-2MOEG4**, (b) keeping in the dark for 5.5 h to reach ***$E_S$*-2MOEG4**, (c) subsequent irradiating with 365 nm light for 10 min to get ***$Z_M$*-2MOEG4**, (d) finally keeping in the dark for 7.2 h to recover ***$Z_S$*-2MOEG4**.

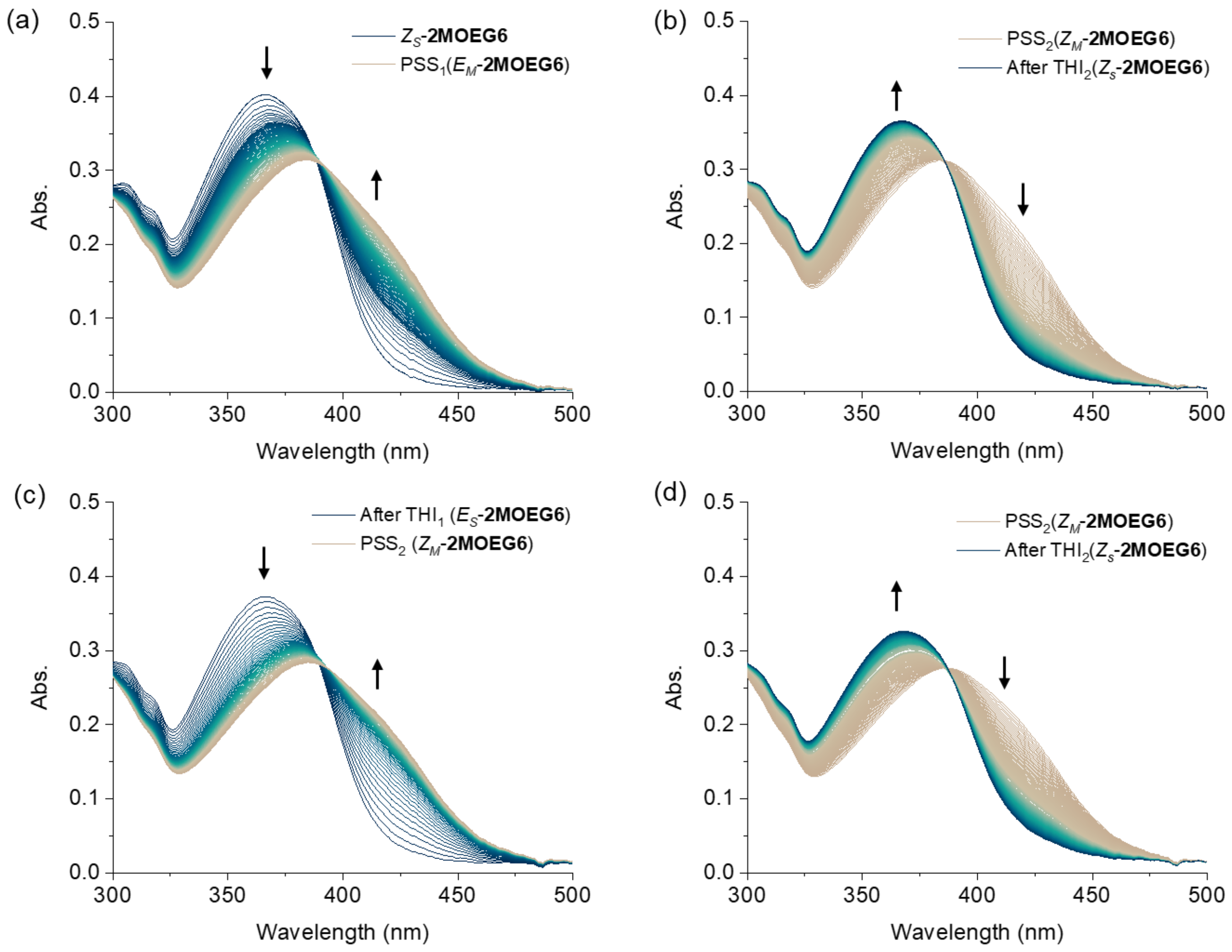


**Supplementary Figure 4. UV-Vis spectroscopy study of the rotation of *Z<sub>S</sub>*-2MOEG4 in water.** UV-Vis absorption spectra of ***Z*$_S$-2MOEG6** (50 μM, 10 ºC) in water (a) upon 365 nm light irradiation for 10 min to get ***E*$_M$-2MOEG6**, (b) keeping in the dark for 2.9 h to reach ***E*$_S$-2MOEG6**, (c) subsequent irradiating with 365 nm light for 10 min to get ***Z*$_M$-2MOEG6**, (d) finally keeping in the dark for 3.6 h to recover ***Z*$_S$-2MOEG6**.

## 2. Transmittance measurement

Transmittance measurements were recorded on a Jasco V-750 spectrometer in a quartz cuvette with a 1 cm path length. The transmittance of samples was monitored at the wavelength of 500 nm. Temperature-dependent transmittance measurements were performed with a heating rate of 1°C/min. Phase transition temperatures measured from transmittance were used to plot the phase diagram.

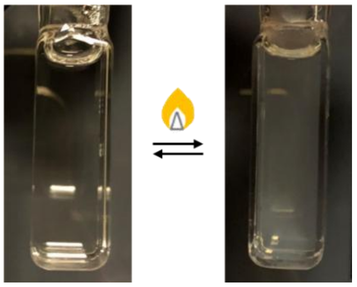

**Supplementary Figure 5.** Images of an aqueous solution of ***Z*$_S$-2MOEG4** before and after heating in the cuvette. Samples were used for temperature-dependent transmittance measurements.

The LLPS behavior of aged supramolecular assemblies was investigated using temperature-dependent transmittance measurements. At the same concentration, the critical temperature ($T_c$) of the aged ***Z*$_S$-2MOEG4**

assembly solution was found to decreased by 4 °C (Supplementary Fig. 6). Cryo-TEM measurements reveal that the length of worm-like micelles grows from ~20 nm in the fresh state to several micrometers after aging. The decrease of $T_c$ may be attributed to the increased length of supramolecular polymers upon aging. This observation is consistent with trends seen in covalent polymers exhibiting LCST behavior, where increasing molecular weight leads to a lower LCST (Chem. Soc. Rev., 2013, 42, 7468).

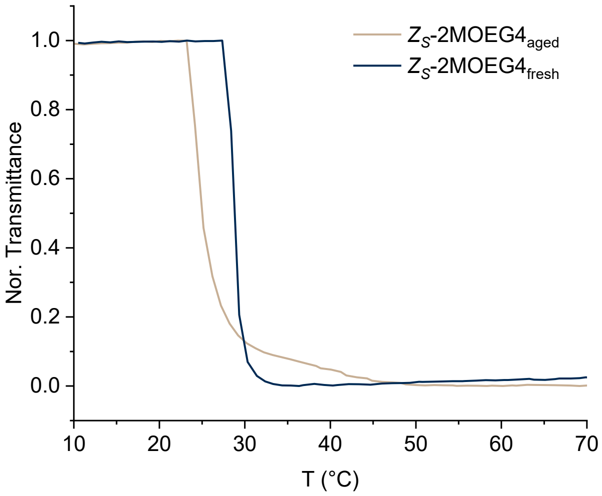


**Supplementary Figure 6.** Transmittance of aqueous solutions of fresh and aged ***Z<sub>S</sub>*-2MOEG4** (0.3 mg/mL) upon heating.

### 3. Cryogenic transmission electron microscopy (Cryo-TEM) study

A water solution of ***$Z_S$*-2MOEG4** (0.5 mg/mL) was treated with ultrasound to be dispersed and kept in the dark at room temperature (RT) for 1 h (Fig. 4j). The sample was first irradiated with 365 nm light for 10 min to get ***$E_M$*-2MOEG4** in a quartz cuvette with 1 mm path length (Fig. 4l). The obtained sample was kept in the dart at room temperature for 60 min to reach ***$E_S$*-2MOEG4** state (Fig. 4p). The sample was subsequent irradiating with 365 nm light for 10 min to get ***$Z_M$*-2MOEG4** (Fig. 4n). Finally, the sample was kept in the dark at 25 ºC for 60 min to recover ***$Z_S$*-2MOEG4**. In each state, a few microliters of sample solution were placed on holey carbon-coated copper grids (Quantifoil 3.5/1, Quantifoil Micro Tools, Jena, Germany). Grids with samples were vitrified in liquid ethane (Vitrobot, FEI, Eindhoven, The Netherlands) and transferred to a FEI Tecnai T20 cryo-electron microscope operating at 200 keV Images were recorded under low-dose conditions with a slow scan CCD camera. ***$Z_S$*-2MOEG6** and aged ***$Z_S$*-2MOEG4** solutions were measured using the same method.

### 4. Dynamic light scattering (DLS) measurements

The water solution of motor amphiphiles (0.5 mg/mL) was treated with ultrasound for 1 min to be dispersed and stabilized at room temperature for 1 h. The aqueous sample solution was placed in a plastic cuvette. DLS measurements are conducted on Zetasizer Ultra equipment with a fluorescence filter using He-Ne laser (633 nm). All the setups were calibrated before measurements. For each temperature, five measurements were performed after equilibrium for 300 s. Data were analyzed in the ZS XPLORER software, assuming a refractivity index of 1.56. The size of assemblies was determined following the number distribution. Temperature-dependent measurements were taken every 5 ºC and the sample was held at each temperature for 5 minutes to reach equilibrium. The measurements for the aging effect on the assemblies are conducted at room temperature.

As standard DLS assumes spherical particle morphology, there are inherent limitations when analyzing elongated structures such as fibers. The reported hydrodynamic diameter reflects an equivalent scattering radius based on Brownian motion, rather than the actual length of supramolecular fibers. The primary purpose of the DLS measurement was to qualitatively compare the assemblies before and after aging, rather than to precisely define fiber dimensions.

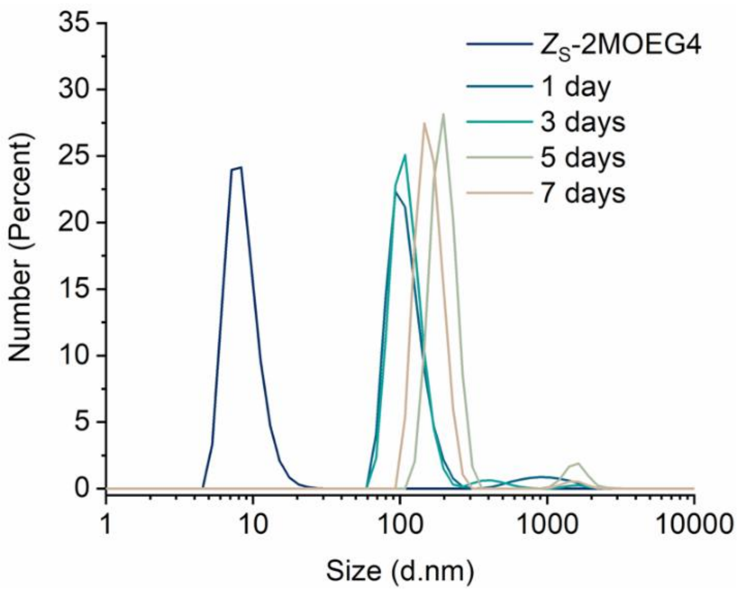


**Supplementary Figure 7.** Time-dependent DLS data of ***Z<sub>S</sub>*-2MOEG4** in water.

Since ***Z<sub>S</sub>*-2MOEG4** formed fibers with a uniform diameter of 5 nm and micrometers length after aging (Fig. 2h). The DLS data shows two peaks of the aged ***Z<sub>S</sub>*-2MOEG4** solution, which may be due to the linear nature of the fibers.

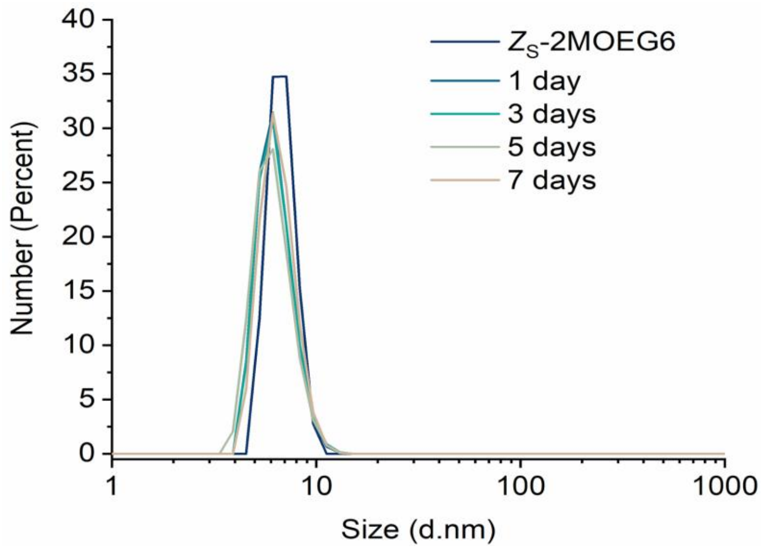


**Supplementary Figure 8.** Time-dependent DLS data of ***Z<sub>S</sub>*-2MOEG6** in water.

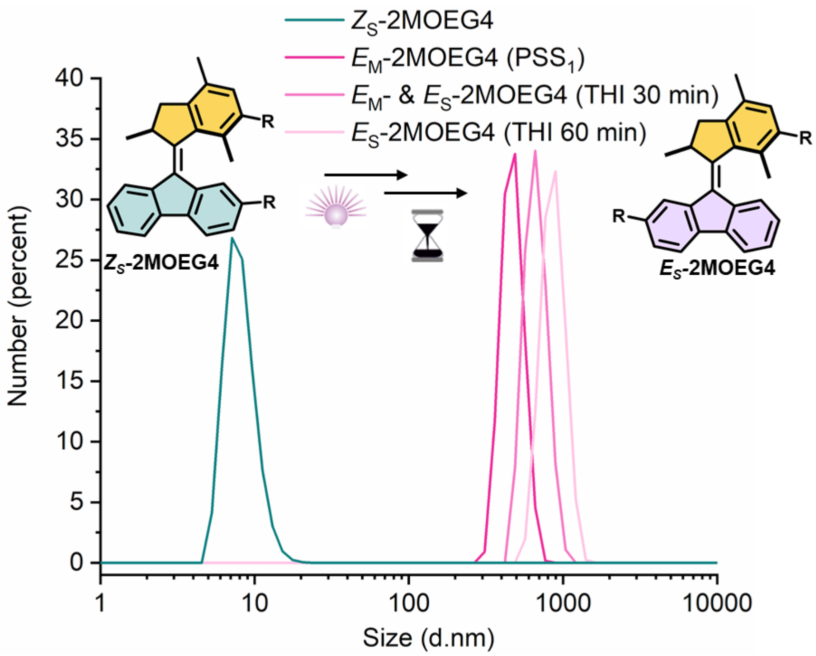


**Supplementary Figure 9.** DLS data of ***Zs*-2MOEG4** before and after irradiation and after subsequent thermal helix inversion in aqueous solutions.

DLS studies revealed that the hydrodynamic diameter of the droplets formed by aged ***Zs*-2MOEG4** assemblies is around 400 nm upon phase separation (Supplementary Fig. 10). Moreover, once the temperature decreases below $T_c$, the assemblies reverted to their original size.

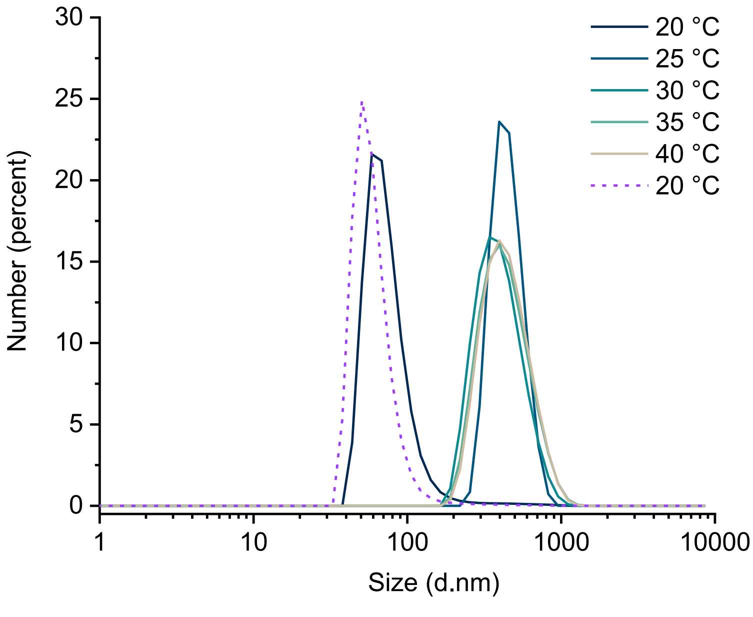


**Supplementary Figure 10.** Time-dependent DLS data of aged ***Zs*-2MOEG4** assembly in water.

### 5. Fourier transform infrared (FTIR) spectroscopy study

FTIR spectra were recorded using a PerkinElmer Spectrum Two FTIR spectrometer. Samples were prepared in water to form the supramolecular assemblies and were measured after drying from solution. Spectra were collected in the range of 800–4000 cm⁻¹ over 64 scans.

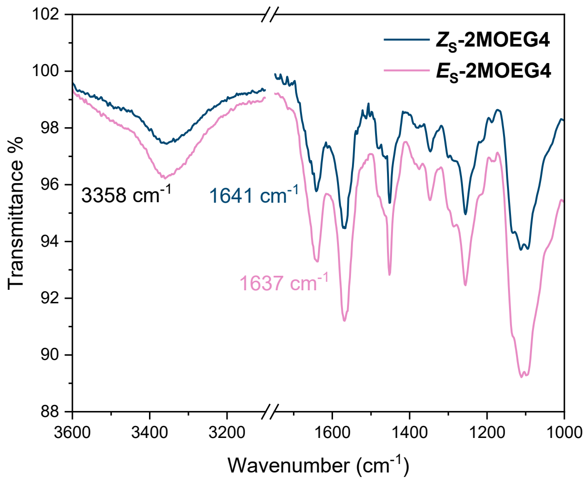


**Supplementary Figure 11.** FTIR spectra of supramolecular assemblies formed by ***Z*s-2MEG4** and ***E*s-2MEG4.** Samples were characterized after drying from aqueous solution.

### 6. Nile Red fluorescence assay

A stock solution of Nile Red (250 µM) was prepared in methanol and freshly diluted 1:200 with Milli-Q water to obtain a final concentration of 2.5 µM. Stock solutions of motor amphiphiles (100 µM) were prepared by dissolving the compounds in Milli-Q water, followed by 30 seconds of ultrasonic treatment. These stock solutions were further diluted to the desired concentrations with Milli-Q water. Equal volumes of the aqueous Nile Red solution were added to each sample. All solutions were aged for 1 hour in a 96-well microplate. Fluorescence emission spectra were recorded using a Tecan Spark plate reader, with an excitation wavelength of 550 nm and emission measured from 580 to 700 nm. The Δλ values were averaged over three replicates.

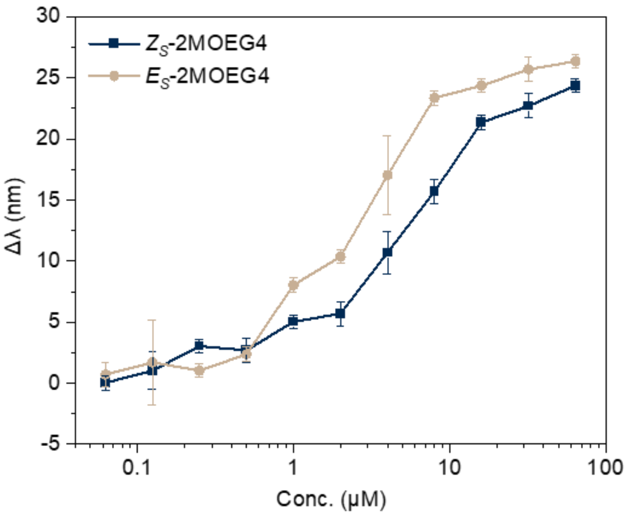


**Supplementary Figure 12.** The blueshift of Nile Red fluorescence as a function of ***Z*s-2MOEG4** and ***E*s-2MOEG4** concentration. N = 3. Error bars denote the standard deviation (SD).

## 7. Confocal laser scanning microscopy (CLSM) study

The water solution of **$Z_S$-2MOEG4** (0.5 mg/mL) was treated with ultrasound for 1 min to be dispersed and stabilized at room temperature (RT) for 1 h. The light irradiation procedure and thermal helix inversion were the same as for the cryo-TEM study. Samples were dyed with 0.5 μM of Nile Red. 9.2 μL of each sample was loaded into a 120 μm thick sample chamber consisting of two coverslips and an imaging spacer in the center (Grace Bio-Labs SecureSeal Imaging Spacer, diameter: 9 mm) and measured via Leica TCS SP8 equipped with a 63×1.2 (water immersion) numerical aperture objective. A laser of 552 nm was used as the excited light source, and the emission was recorded at the wavelength range of 580-750 nm.

Temperature-controlled CLSM studies were performed using a VaHeat (Interherence) system. 2 μL of sample solution was loaded into a glass capillary (0.2×3×15 mm) for temperature-variable measurements. The glass capillary was placed on the VaHeat microscopy-compatible stage and temperature was controlled *in situ* on Leica TCS SP8 equipped with a 40×1 numerical aperture objective.

Movies are recorded on Leica TCS SP8 equipped with a 40×1 numerical aperture objective and processed in ImageJ software.

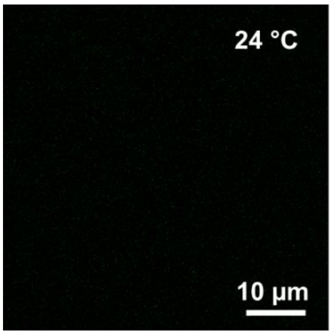


**Supplementary Figure 13.** CLSM image of homogenous solution of **$Z_S$-2MOEG4** at room 24 °C. No droplets were found. The result was consistent with DLS study.

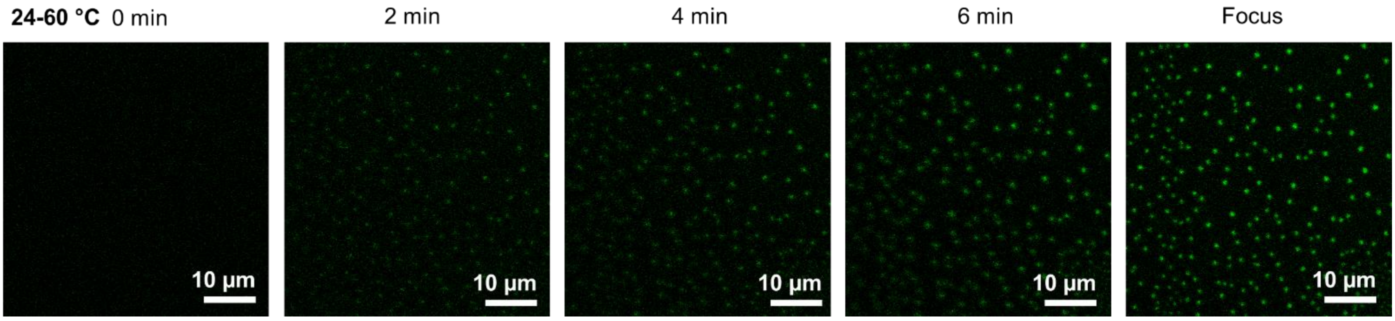


**Supplementary Figure 14.** Time-lapse CLSM images of homogenous solution of **$Z_S$-2MOEG4** upon heating to 60 °C, in which droplets generated again.

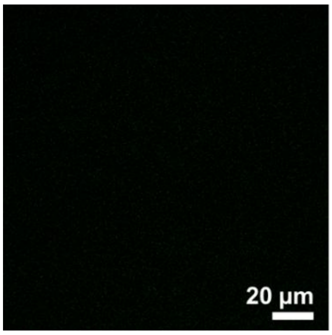


**Supplementary Figure 15.** CLSM image of homogenous solution of the recovered **$Z_S$-2MOEG4**.

## 8. Fluorescence recovery after photobleaching (FRAP)

The water solution of $Z_S$**-2MOEG4** (0.5 mg/mL) was dyed with 0.5 μM of Nile Red. Two images were taken before bleaching at an imaging power of 1.0%, using a laser of 552 nm as the excitation light source, recorded at the wavelength range of 580-750 nm. Subsequently, selected circular areas (regions of interest, ROIs) with diameters ranging from 5.0 to 8.0 μm were bleached for 10 cycles (1.290 s per cycle) at 95% of the power with the 552 nm laser. The following images were automatically captured every 1.29 seconds at 1.0% power. Five measurements were conducted for each sample. The fluorescence intensities of ROIs were extracted by built-in software LAS X.

## 9. Eyring analysis on the thermal helix inversion of molecular motors

Eyring analysis is described in detail in the Methods section of the main text.

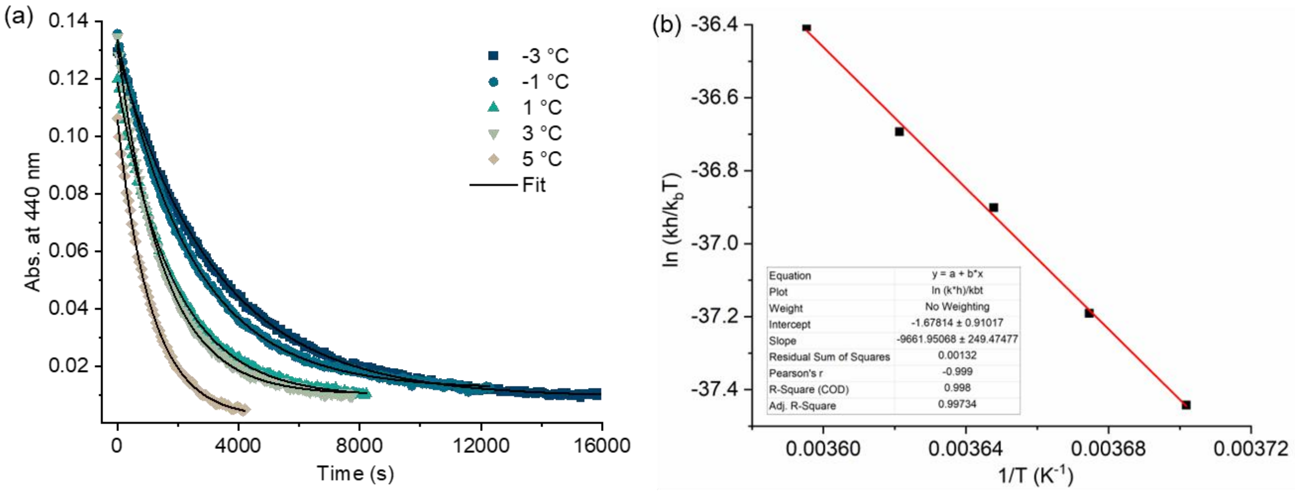


**Supplementary Figure 16. Eyring analysis on the THI of $Z_M$-2MOEG6 in MeOH.** (a) Absorption at 440 nm as a function of time during the THI of $Z_M$**-2MOEG6** (50 μM) in MeOH at different temperatures. (b) Plot of ln($kh/K_BT$) versus $1/T$.

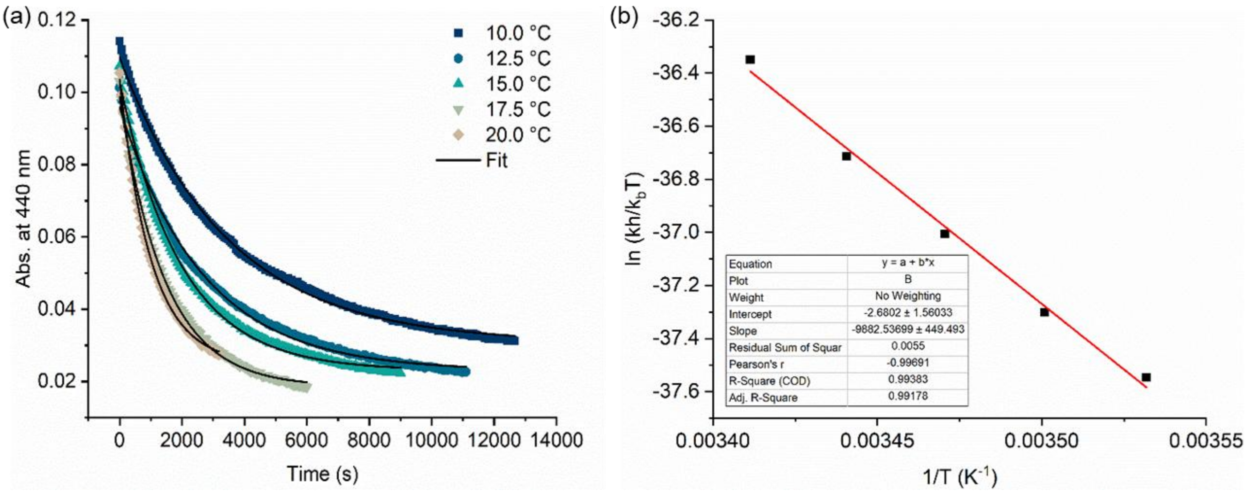


**Supplementary Figure 17. Eyring analysis on the THI of $Z_M$-2MOEG6 in water.** (a) Absorption at 440 nm as a function of time during the THI of $Z_M$**-2MOEG6** (50 μM) in water at different temperatures. (b) Plot of ln($kh/K_BT$) versus $1/T$.

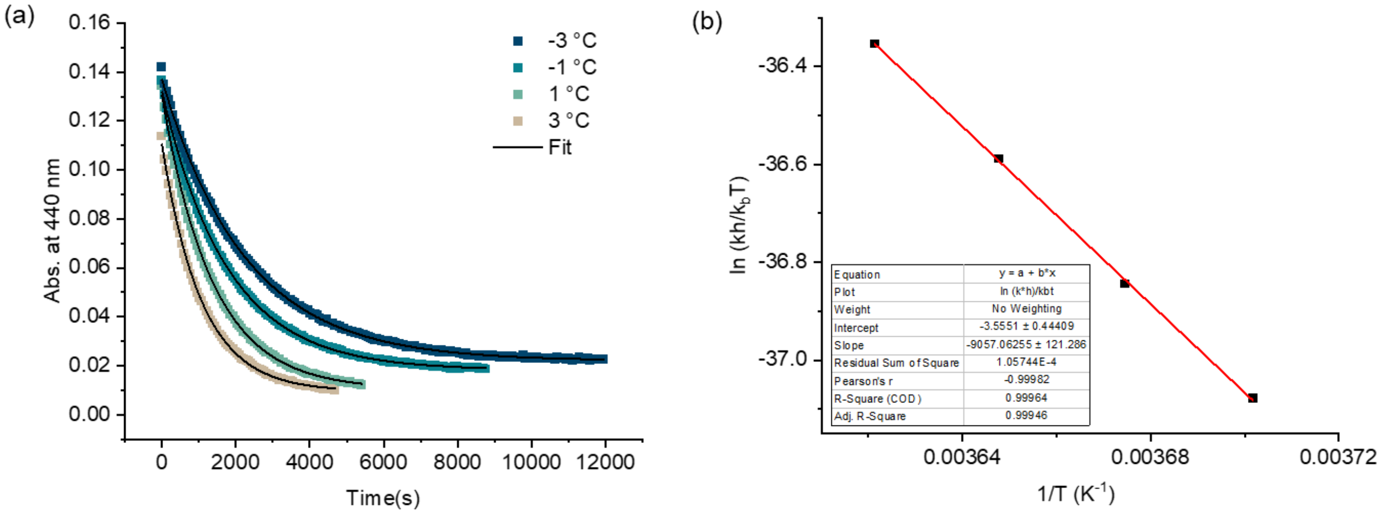


**Supplementary Figure 18. Eyring analysis on the THI of *E$_M$*-2MOEG4 in MeOH.** (a) Absorption at 440 nm as a function of time during the THI of ***E$_M$*-2MOEG4** (50 µM) in MeOH at different temperatures. (b) Plot of ln(*kh*/*K$_B$T*) versus 1/*T*.

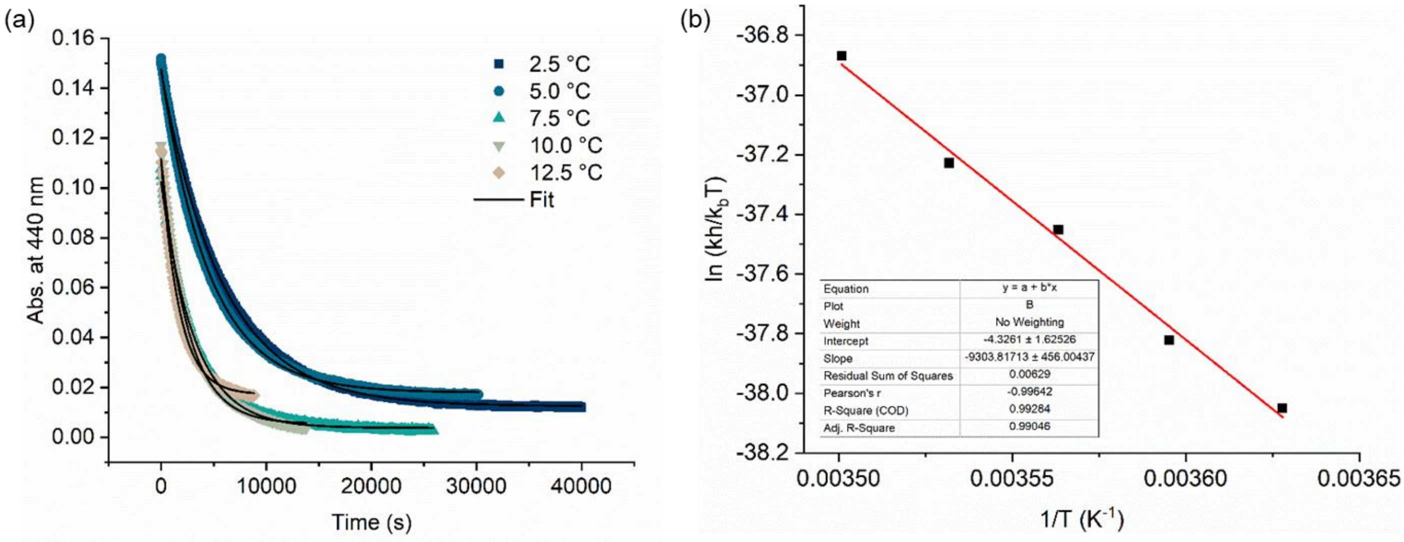


**Supplementary Figure 19. Eyring analysis on the THI of *E$_M$*-2MOEG4 in water.** (a) Absorption at 440 nm as a function of time during the THI of ***E$_M$*-2MOEG4** (50 µM) in water at different temperatures. (b) Plot of ln(*kh*/*K$_B$T*) versus 1/*T*.

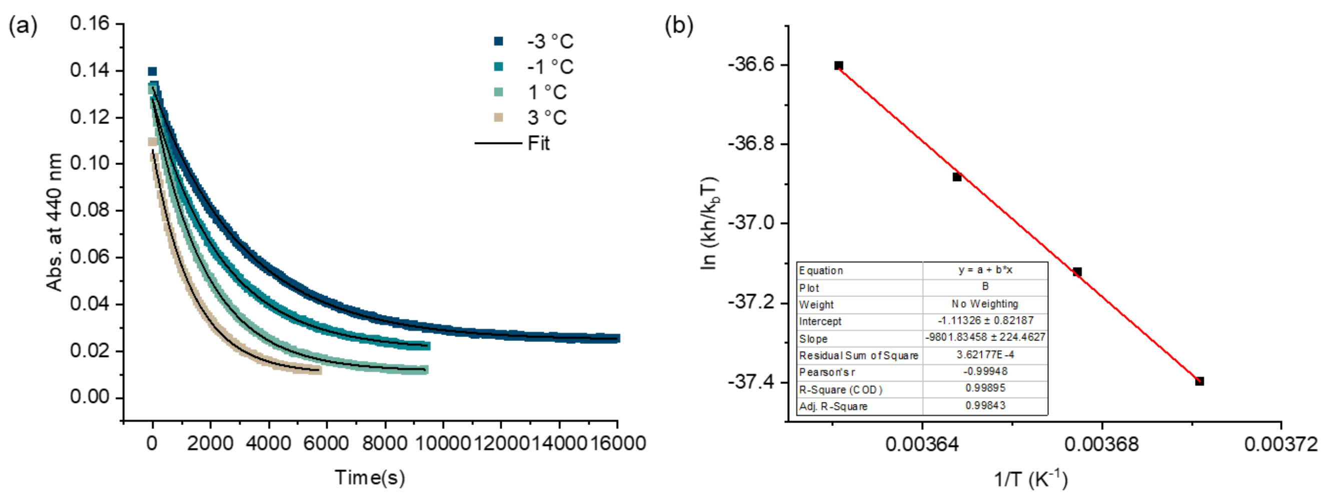


**Supplementary Figure 20. Eyring analysis on the THI of $Z_M$-2MOEG4 in MeOH.** (a) Absorption at 440 nm as a function of time during the THI of ***$Z_M$*-2MOEG4** (50 μM) in MeOH at different temperatures. (b) Plot of ln($kh/K_BT$) versus 1/$T$.

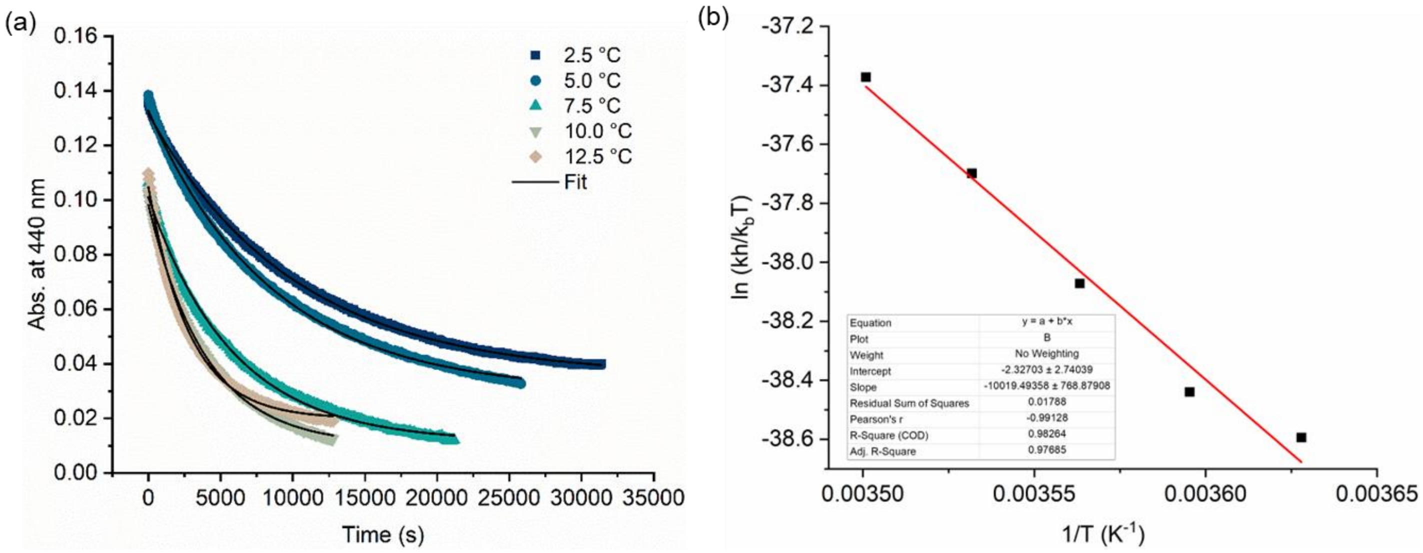


**Supplementary Figure 21. Eyring analysis on the THI of $Z_M$-2MOEG4 in water.** (a) Absorption at 440 nm as a function of time during the THI of ***$Z_M$*-2MOEG4** (50 μM) in water at different temperatures. (b) Plot of ln($kh/K_BT$) versus 1/$T$.

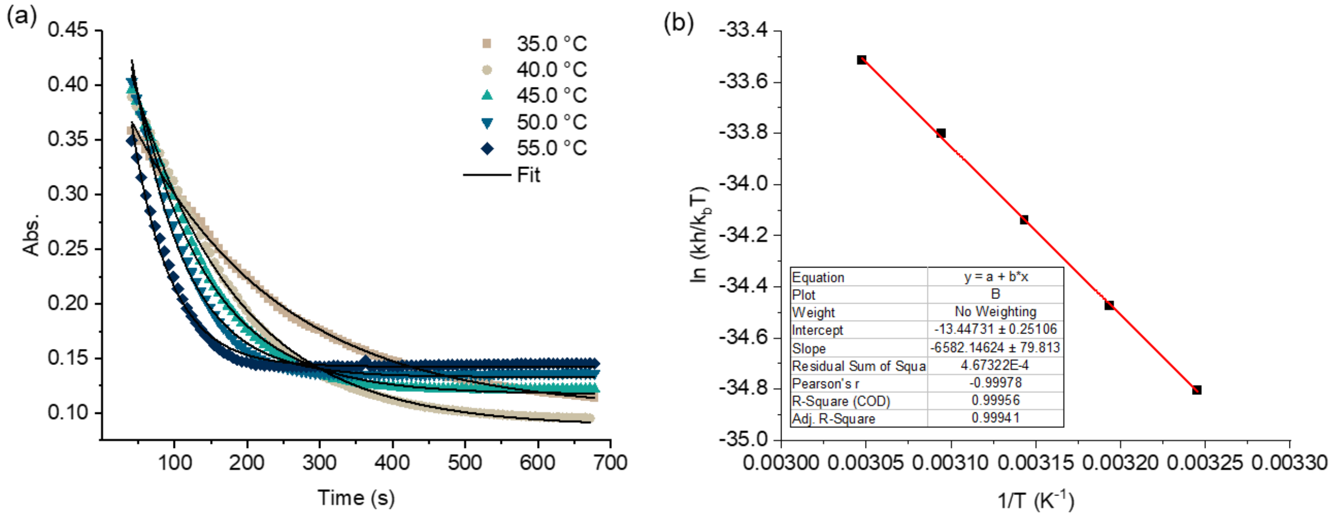


**Supplementary Figure 22. Eyring analysis on the THI of $E_M$-2MOEG4 after phase separation in water.** (a) Absorption at 440 nm subtracting the absorption at 500 nm as a function of time during the THI of $\boldsymbol{E_M}$**-2MOEG4** (90 μM) in water after phase separation (in the droplets) at different temperatures. (b) Plot of $\ln(kh/K_BT)$ versus $1/T$.

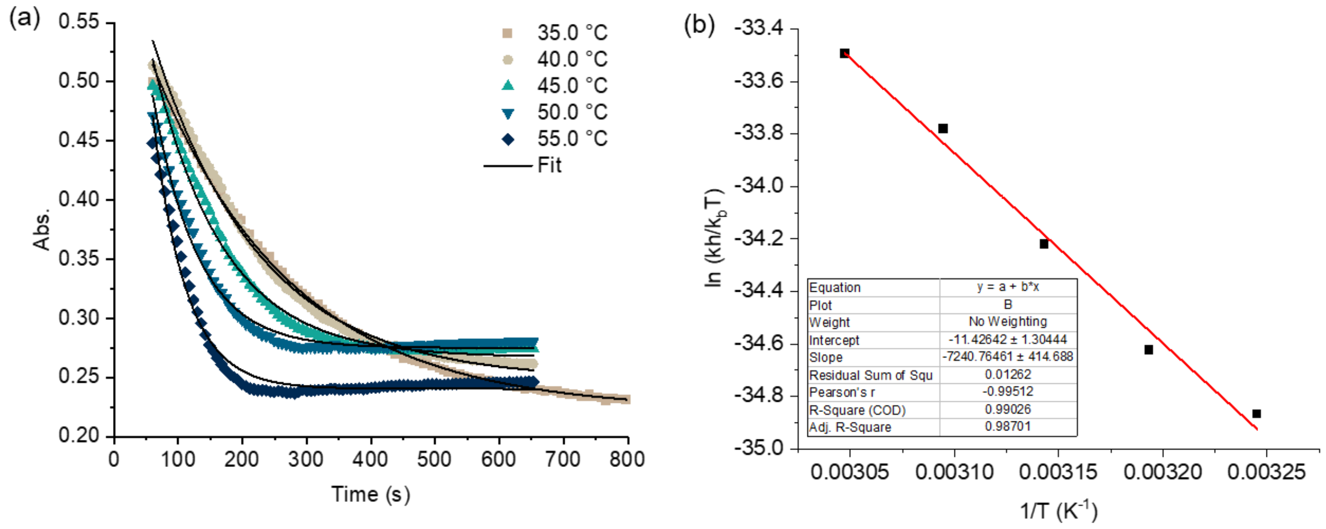


**Supplementary Figure 23. Eyring analysis on the THI of $Z_M$-2MOEG4 after phase separation in water.** (a) Absorption at 440 nm subtracting the absorption at 500 nm as a function of time during the THI of $\boldsymbol{Z_M}$**-2MOEG4** (90 μM) in water after phase separation (in the droplets) at different temperatures. (b) Plot of $\ln(kh/K_BT)$ versus $1/T$.

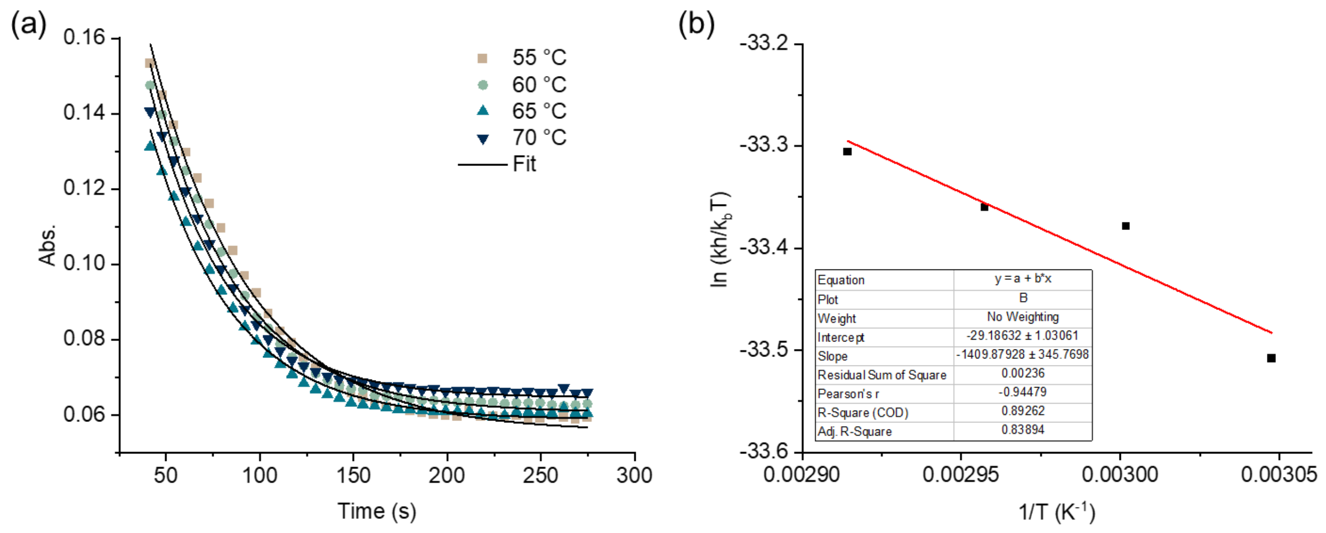


**Supplementary Figure 24. Eyring analysis on the THI of *$E_M$*-2MOEG6 after phase separation in water.** (a) Absorption at 440 nm subtracting the absorption at 500 nm as a function of time during the THI of ***$E_M$*-2MOEG6** (78 µM) in water after phase separation (in the droplets) at different temperatures. (b) Plot of $\ln(kh/K_BT)$ versus $1/T$.

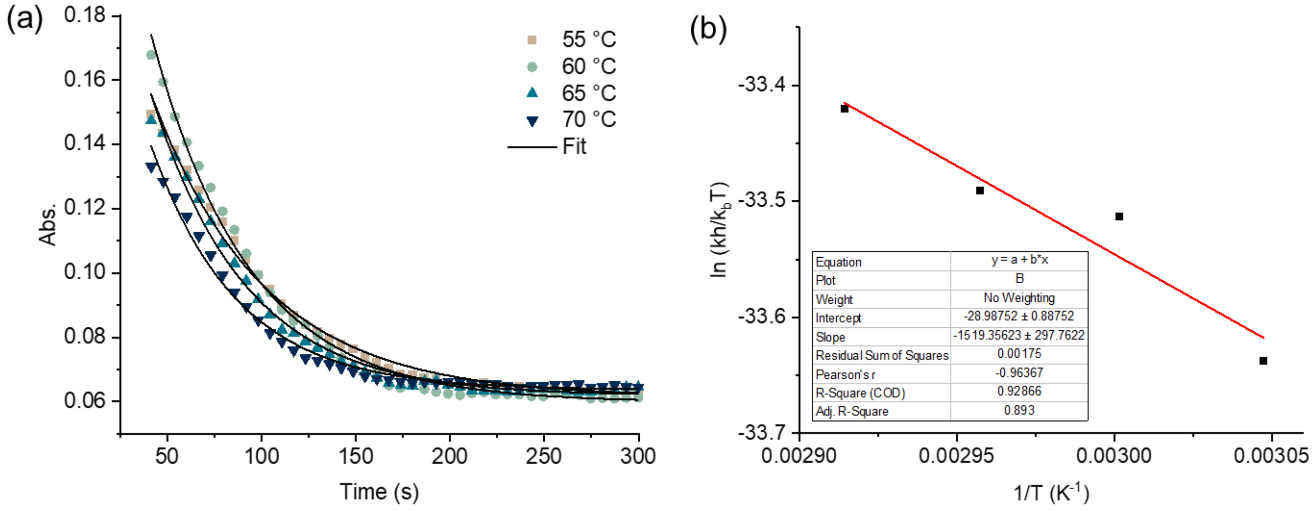


**Supplementary Figure 25. Eyring analysis on the THI of *$Z_M$*-2MOEG6 after phase separation in water.** (a) Absorption at 440 nm subtracting the absorption at 500 nm as a function of time during the THI of ***$Z_M$*-2MOEG6** (78 µM) in water after phase separation (in the droplets) at different temperatures. (b) Plot of $\ln(kh/K_BT)$ versus $1/T$.

**Supplementary Table 1.** Gibbs free energy of activation of THI and the half-life time of metastable motors in water as monomers, in assemblies and in droplets.

| System | $\Delta^{‡}G°$ (kJ mol$^{-1}$)$^{a}$ $E_M$→$E_S$ | $t_{1/2}$ (min)$^{b}$ $E_M$→$E_S$ | $\Delta^{‡}G°$ (kJ mol$^{-1}$)$^{a}$ $Z_M$→$Z_S$ | $t_{1/2}$ (min)$^{b}$ $Z_M$→ $Z_S$ |
|---|---|---|---|---|
| **2MOEG6 as monomer** | 84.3 | 2.0 | 84.4 | 2.1 |
| **2MOEG6 in assemblies** | 87.8 | 8.3 | 88.7 | 12.1 |
| **2MOEG4 as monomer** | 84.0 | 1.7 | 84.2 | 1.9 |
| **2MOEG4 in assemblies** | 87.9 | 8.7 | 89.0 | 13.5 |
| **2MOEG6 in droplets** | 82.8 | 1.1 | 83.3 | 1.3 |
| **2MOEG4 in droplets** | 87.5 | 7.4 | 88.1 | 9.3 |

[a]Determined at 20 °C. [b]Half-life ($t_{1/2}$) defined as ln(2)/$k$ at 20 °C.

## 10. Density functional theory (DFT) calculations

All calculations were performed by the Gaussian 16 package. The ground-state geometries of the ***Z*$_s$-2MOEG4** and ***E*$_s$-2MOEG4** were optimized using density functional theory (DFT) with the B3LYP functional, Grimme's dispersion (B3LYP-D3),[5] and 6-31g (d, p) basis set.[6] Then, frequency calculations at the same level of theory were carried out to identify all the stationary points as minima (zero imaginary frequency). The dipole moments (μ) were extracted based on the optimized structures in Debye units (D). All the calculations were analyzed by the Multiwfn program package.[7]

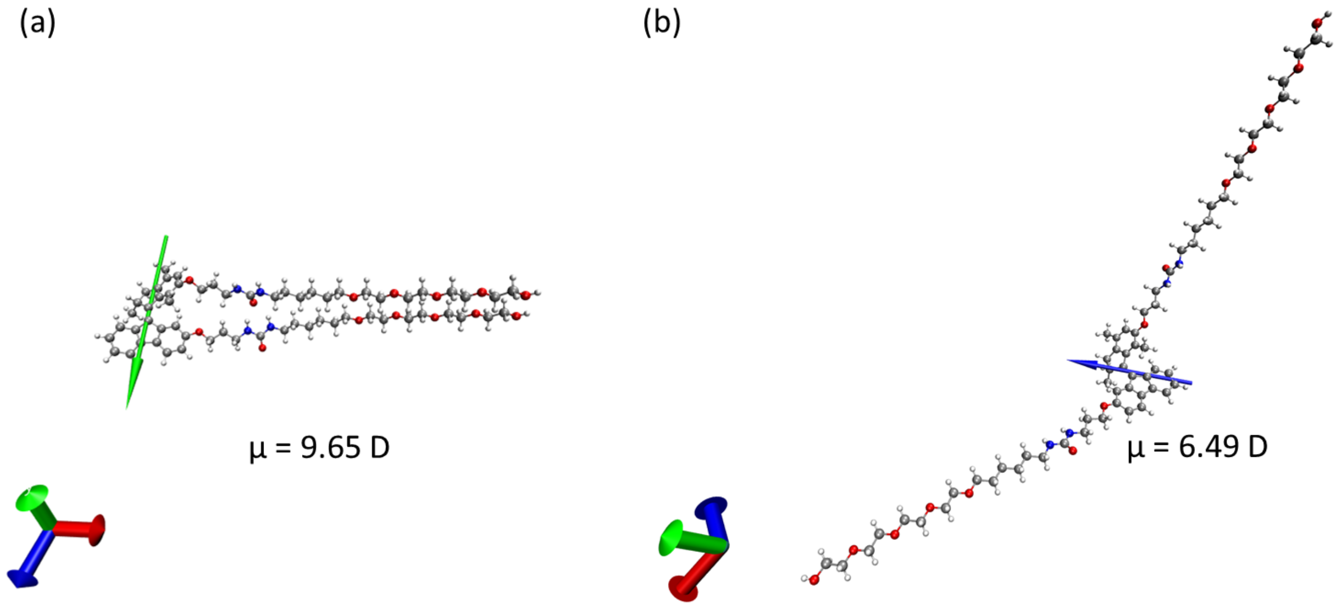


**Supplementary Figure 26. Density functional theory (DFT) calculations of dipole moments.** Optimized structure and dipole moments of (a) ***Z*$_s$-2MOEG4** and (b) ***E*$_s$-2MOEG4**.

# Supplementary Notes

**1**

The observed slight decrease in the energy barrier and half-lifetime of motor rotation after phase separation is likely due to changes in the internal packing environment within the LLPS droplets.

Prior to LLPS, the supramolecular assemblies are stabilized by strong hydrogen bonding and π–π stacking interactions between aromatic motor cores. These interactions lead to relatively tight molecular packing, which restricts the rotational freedom of the motors and contributes to higher activation barriers (Angew. Chem. Int. Ed. 2024, 63, e202319387).

However, LLPS typically occurs at elevated temperatures, which can weaken hydrogen bonding and loosen supramolecular packing. In the condensed phase (droplets), the internal environment is less tightly packed and more dynamic. This provides greater free volume and lower interaction constraints, allowing the molecular motors to rotate more freely. Therefore, the slight reduction in both energy barrier and half-lifetime observed in droplets is consistent with a more flexible, less densely packed local environment at elevated temperatures.

**2**

To indirectly compare the fluidity of the four types of droplets, we investigated the rotational speed of the molecular motors within them. Since the rotation speed of metastable isomer to stable isomer is influenced by the surrounding environment, lower fluidity may result in slower motor rotation. We performed Eyring analysis of THI process of **2MOEG6** (Supplementary Figs. 24 and 25) and compared with that of **2MOEG4** within the droplets (Supplementary Figs. 22 and 23). The critical phase separation temperatures of **2MOEG6** and **2MOEG4** differ; therefore, measurements were conducted at temperatures that allow LLPS for each molecule. For a fair comparison, all half-lifetimes were calculated at 20 °C; however, these values still reflect the motor rotation speed in their measurement environments. The half-lifetimes of ***E*$_M$-2MOEG6** and ***Z*$_M$-2MOEG6** at 20 °C are 1.1 minutes and 1.3 minutes, respectively, which are significantly shorter than those of ***E*$_M$-2MOEG4** and ***Z*$_M$-2MOEG4** (7.4 minutes and 9.3 minutes, respectively). These results suggest **2MOEG6** droplets exhibit higher fluidity than those of **2MOEG4**, possibly due to weaker hydrogen bonding and looser molecular packing at the higher temperatures required for LLPS.

**3**

As the molecular motor used here is a racemic mixture, the supramolecular assemblies are not expected to display chiral order or helicity, which was confirmed by Cryo-TEM analysis. In addition, the CD spectra showed no signal, confirming the absence of supramolecular chirality (Supplementary Fig. 26).

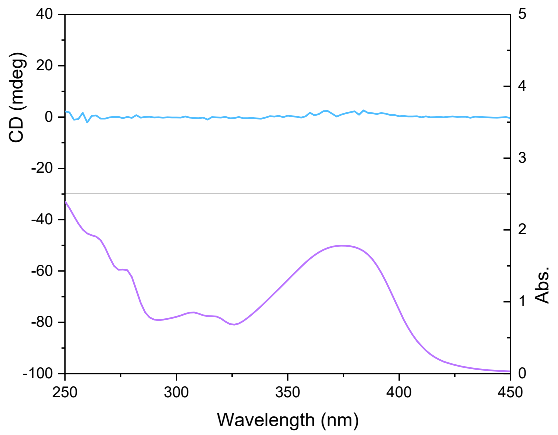


**Supplementary Figure 27.** CD and UV-vis absorption spectra of ***Z*$_S$-2MOEG4** assembly (1mg/mL) in water in a cuvette with 1mm light pathlength.

# Supplementary Figures

## 1. NMR data

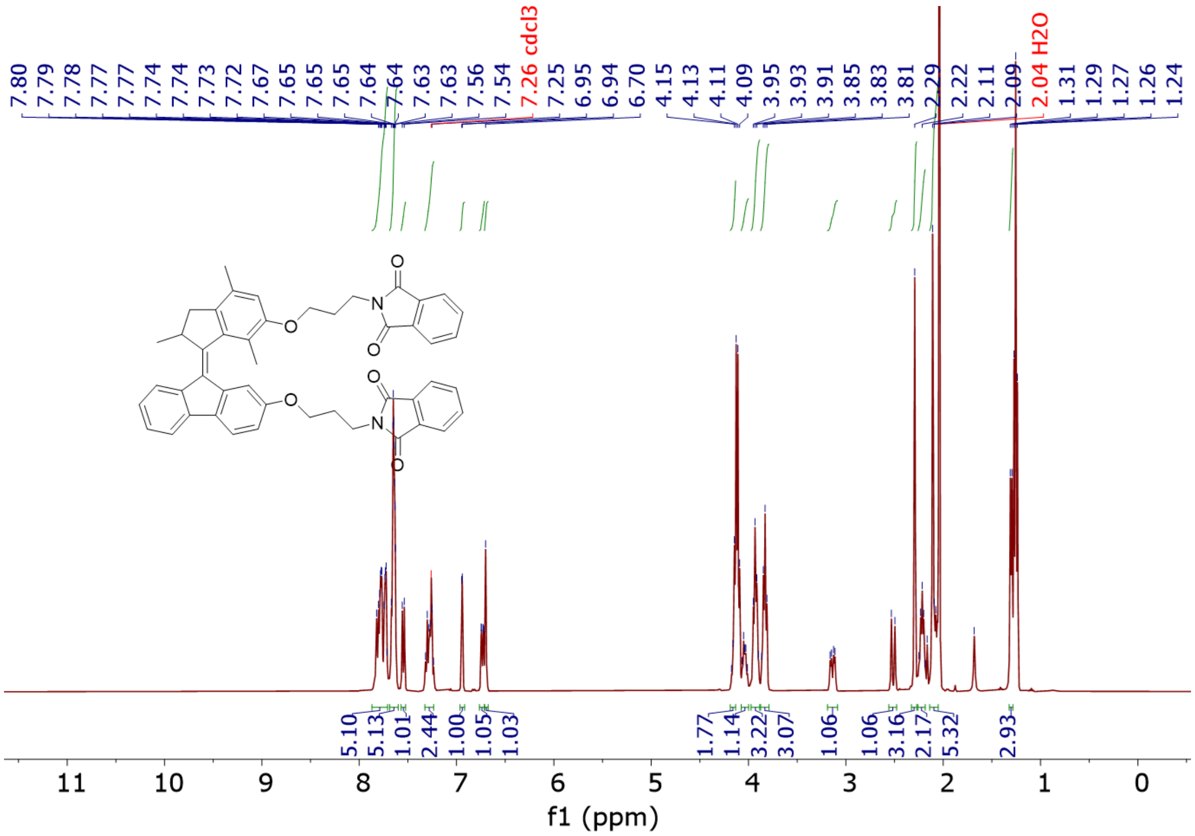


**Supplementary Figure 28.** $^{1}$H NMR spectrum of ***Z<sub></sub>***$Z_S$**-2** ($CDCl_3$, 25 °C, 400 MHz).

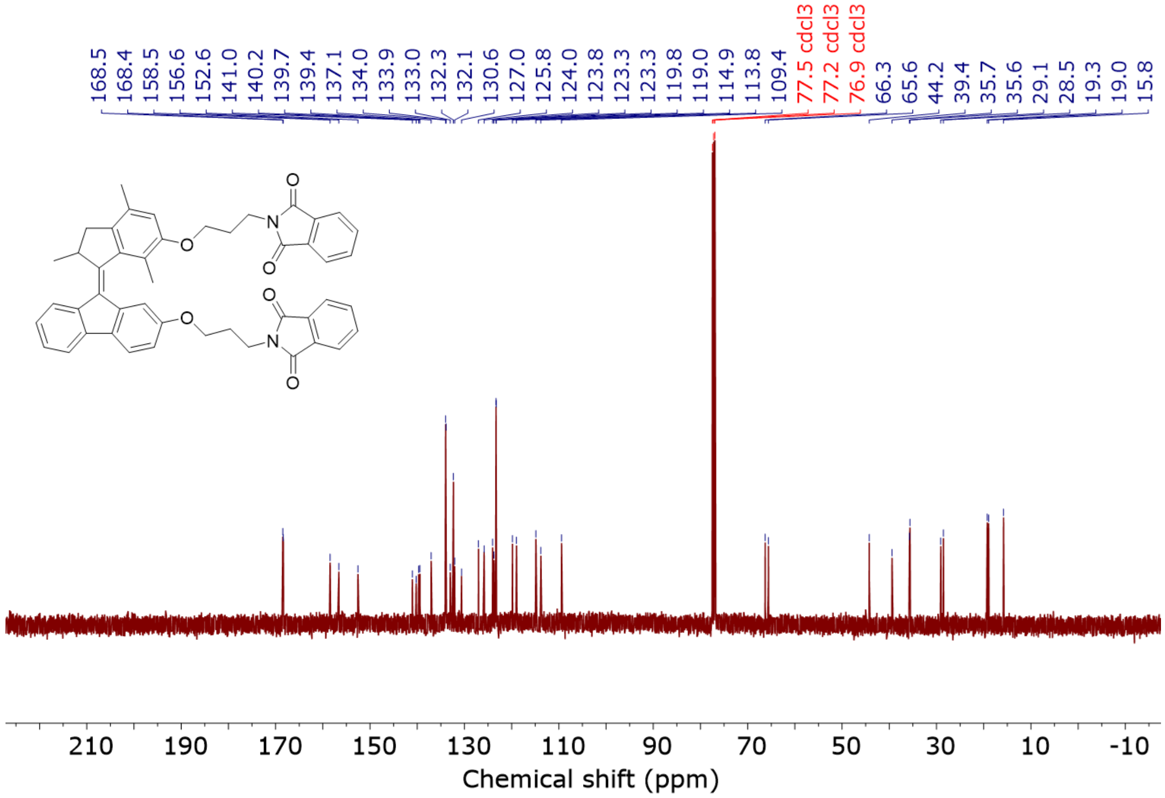


**Supplementary Figure 29.** $^{13}$C NMR spectrum of $Z_S$**-2** ($CDCl_3$, 25 °C, 101 MHz).

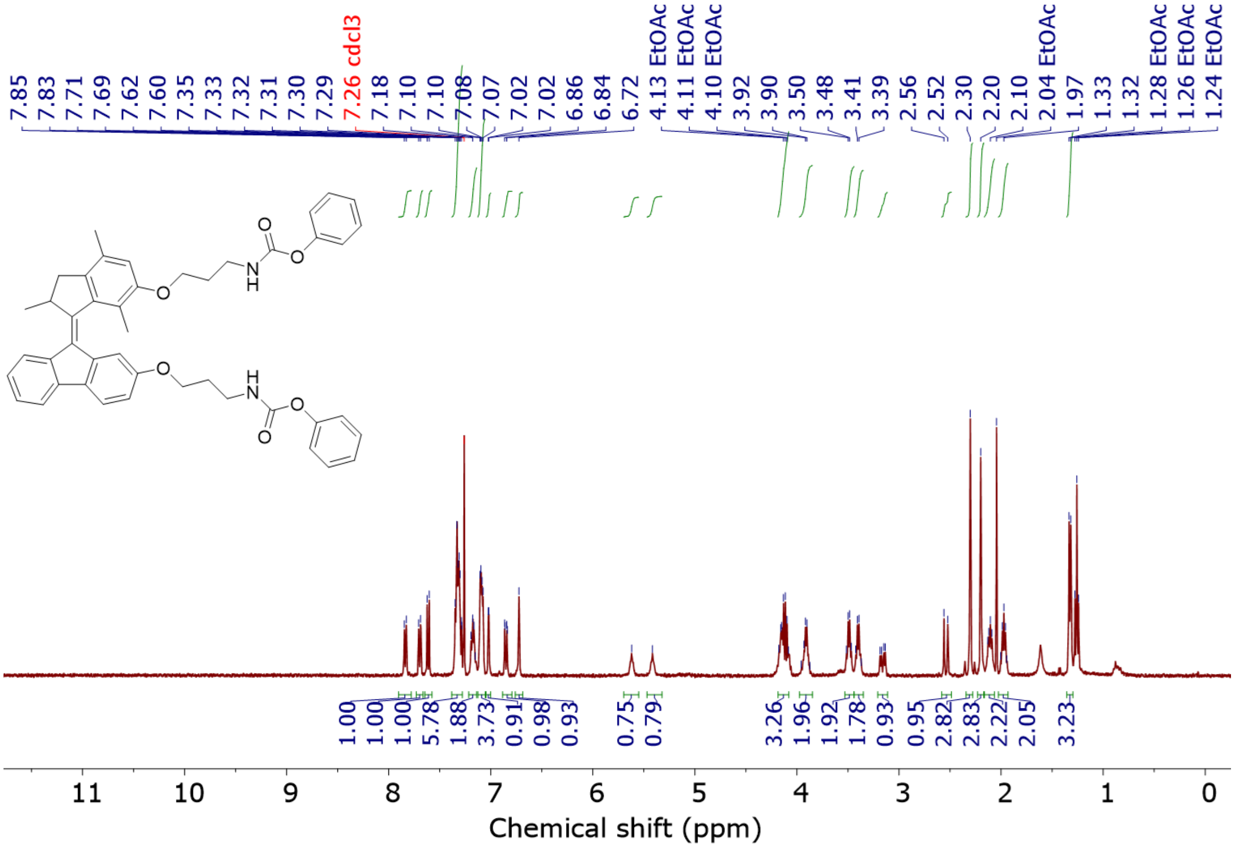


**Supplementary Figure 30.** $^{1}$H NMR spectrum of ***Z*$_S$-3** ($CDCl_3$, 25 °C, 400 MHz).

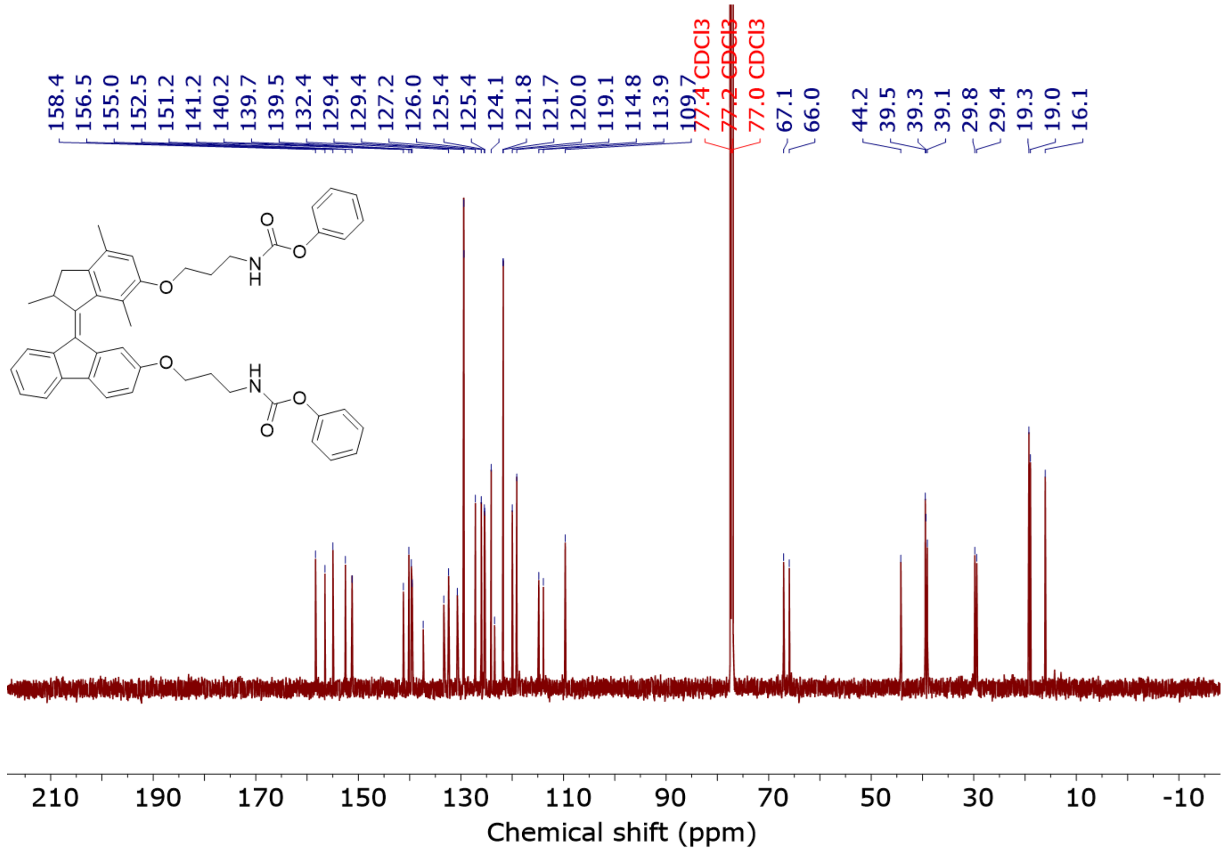


**Supplementary Figure 31.** $^{13}$C NMR spectrum of ***Z*$_S$-3** ($CDCl_3$, 25 °C, 101 MHz).

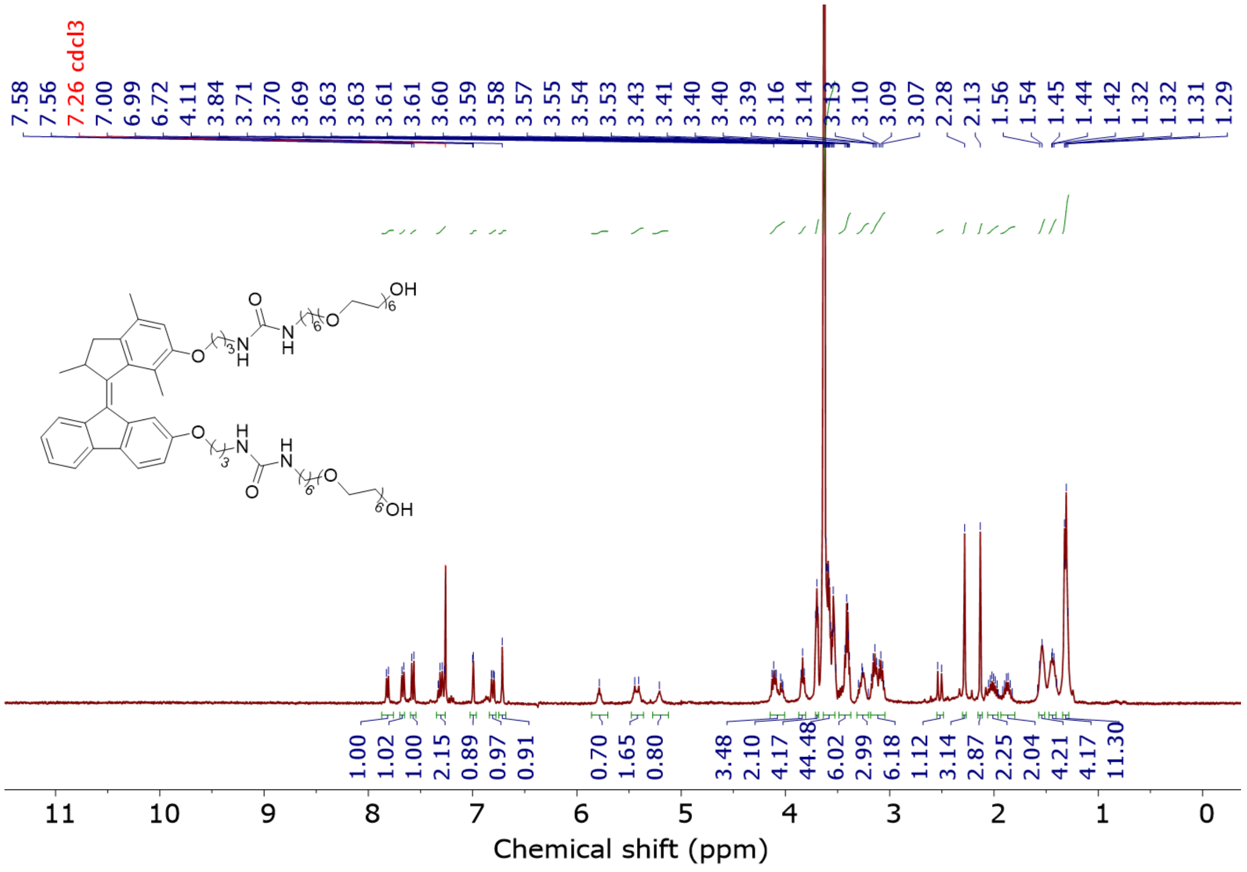


**Supplementary Figure 32.** $^{1}H$ NMR spectrum of ***Z*$_{S}$-2MOEG6** ($CDCl_3$, 25 °C, 400 MHz).

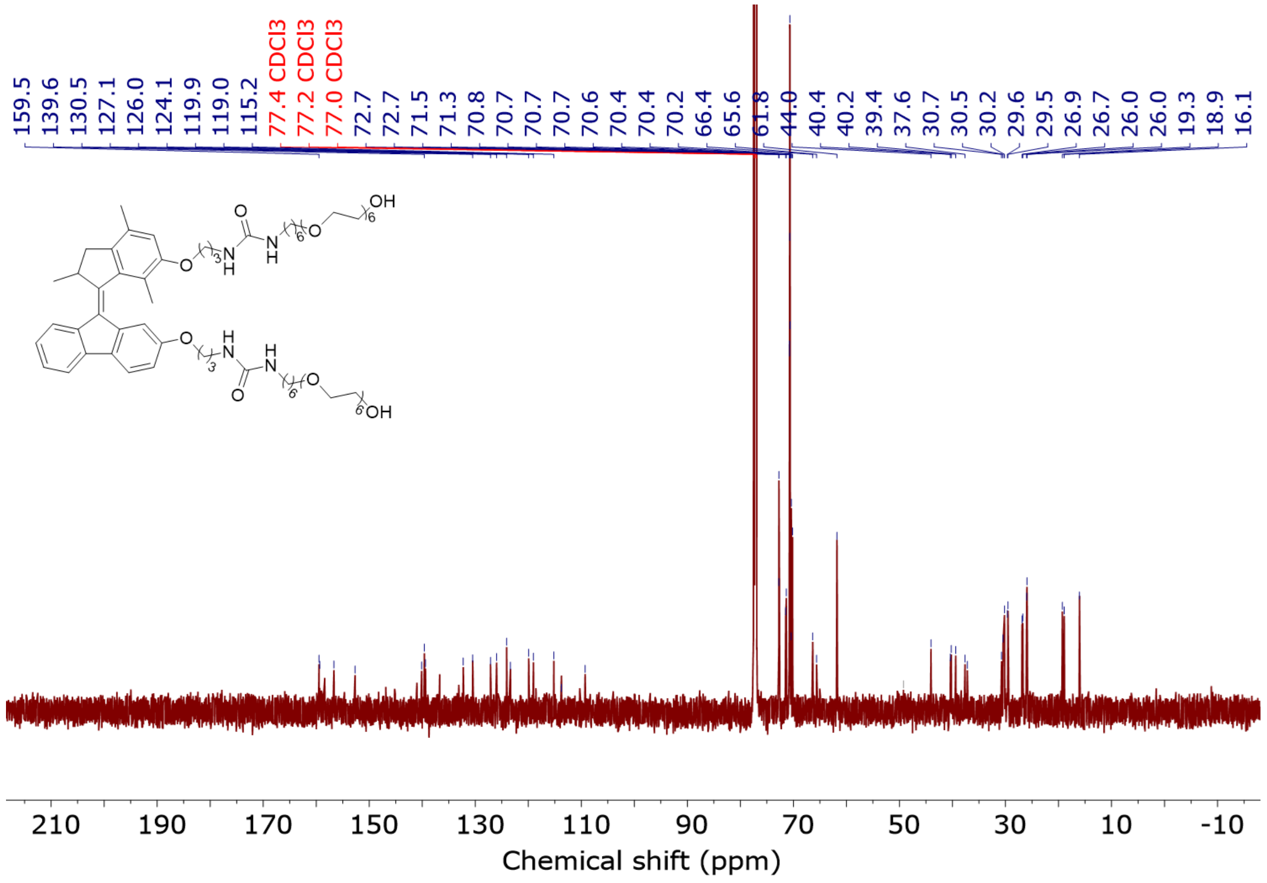


**Supplementary Figure 33.** $^{13}C$ NMR spectrum of ***Z*$_{S}$-2MOEG6** ($CDCl_3$, 25 °C, 151 MHz).

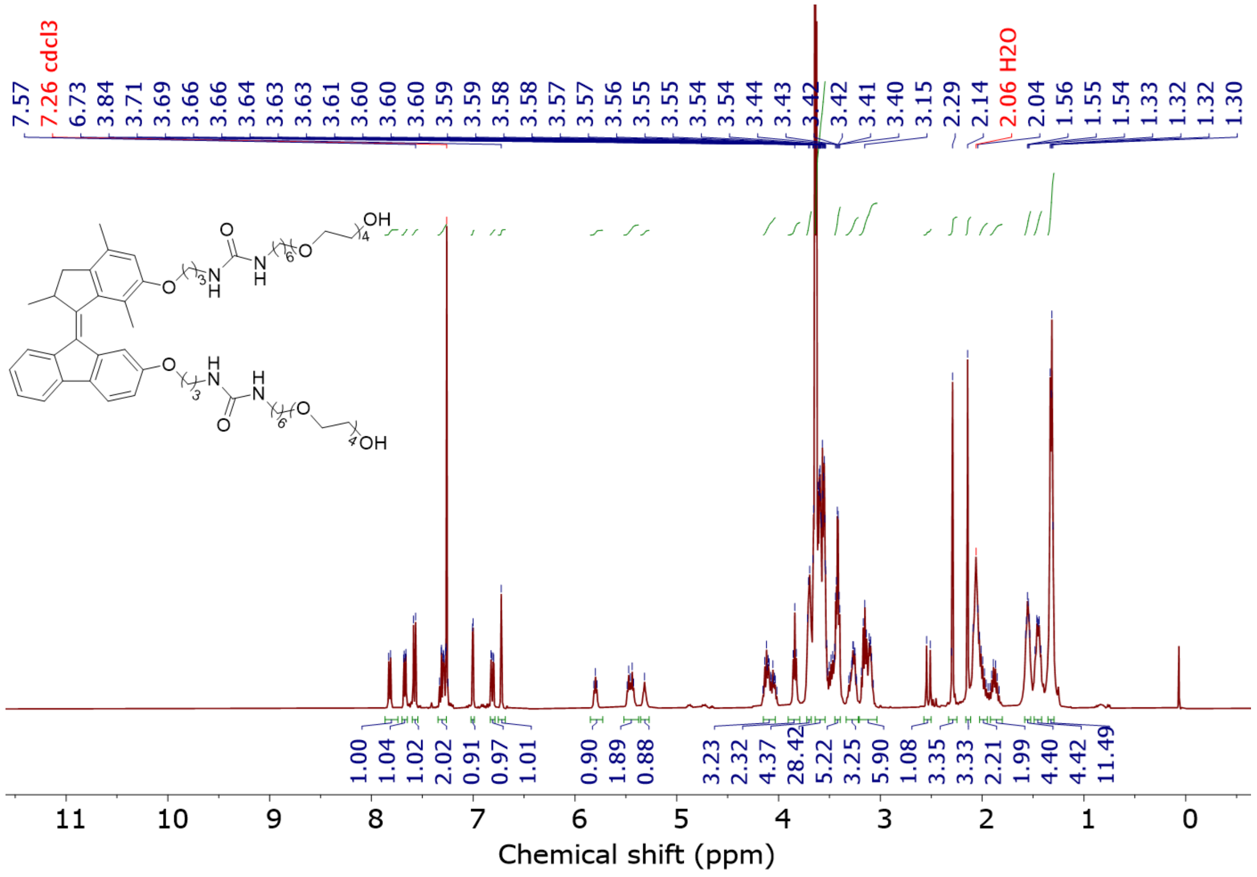


**Supplementary Figure 34.** $^{1}$H NMR spectrum of ***Z$_S$*-2MOEG4** ($CDCl_3$, 25 °C, 400 MHz).

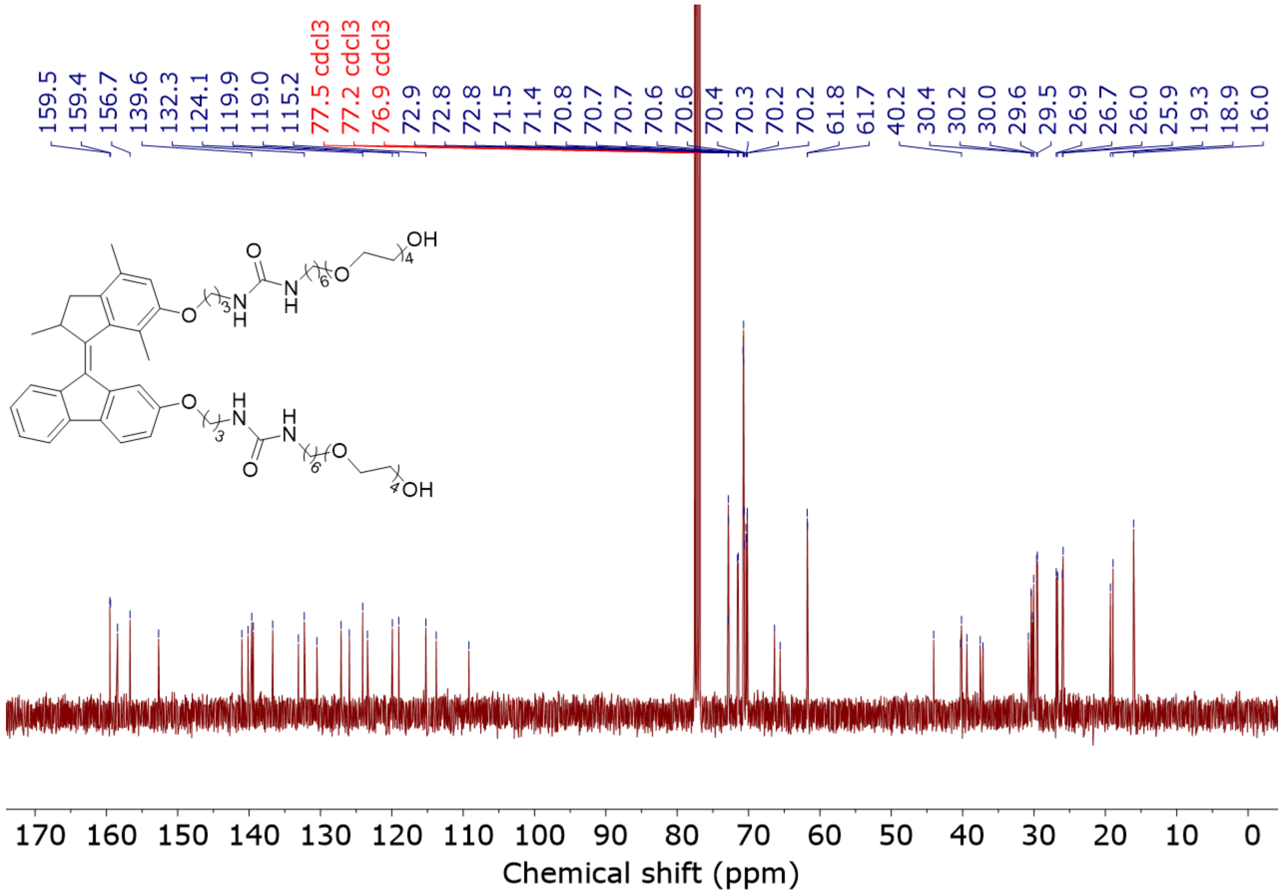


**Supplementary Figure 35.** $^{13}$C NMR spectrum of ***Z$_S$*-2MOEG4** ($CDCl_3$, 25 °C, 101 MHz).

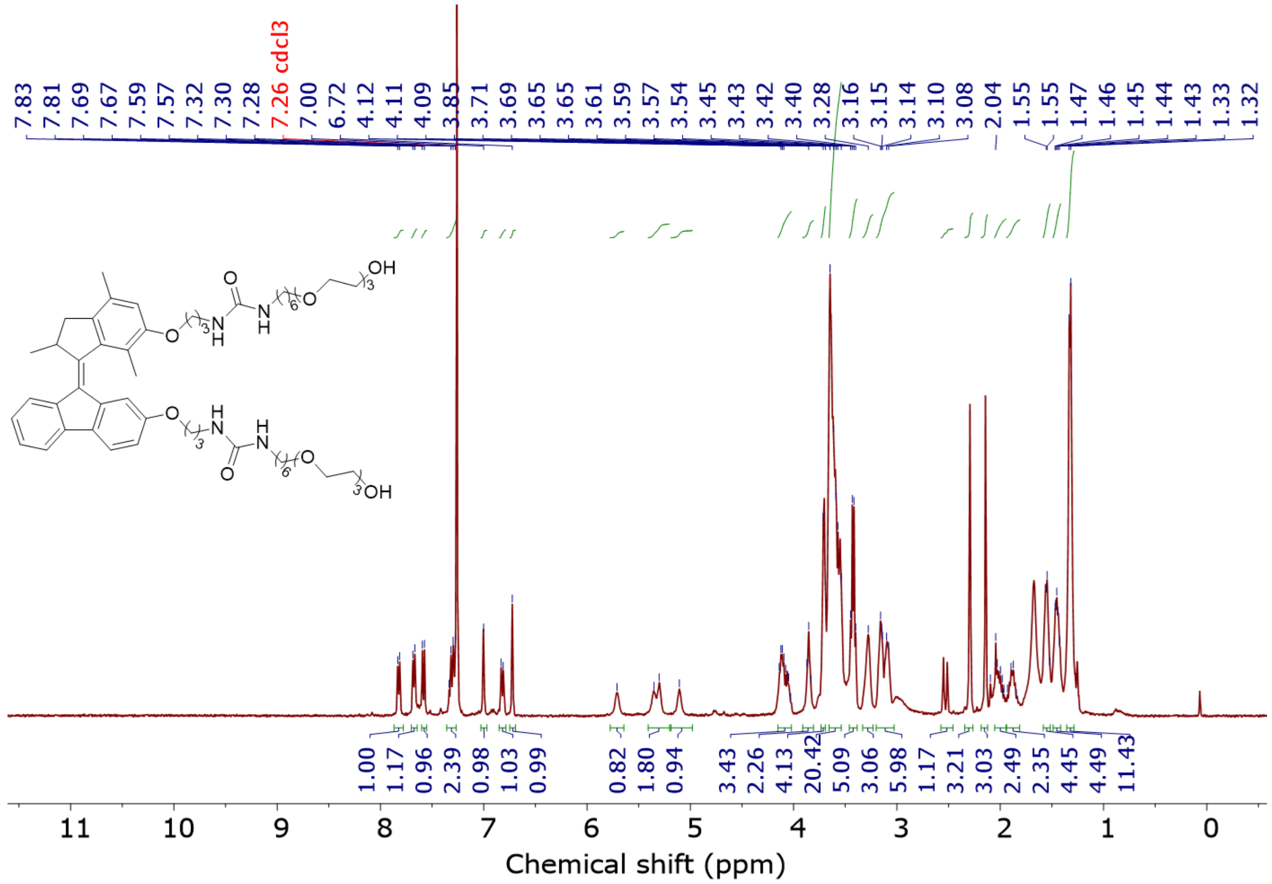


**Supplementary Figure 36.** $^{1}$H NMR spectrum of ***Z<sub>S</sub>*-2MOEG3** (CDCl$_3$, 25 °C, 400 MHz).

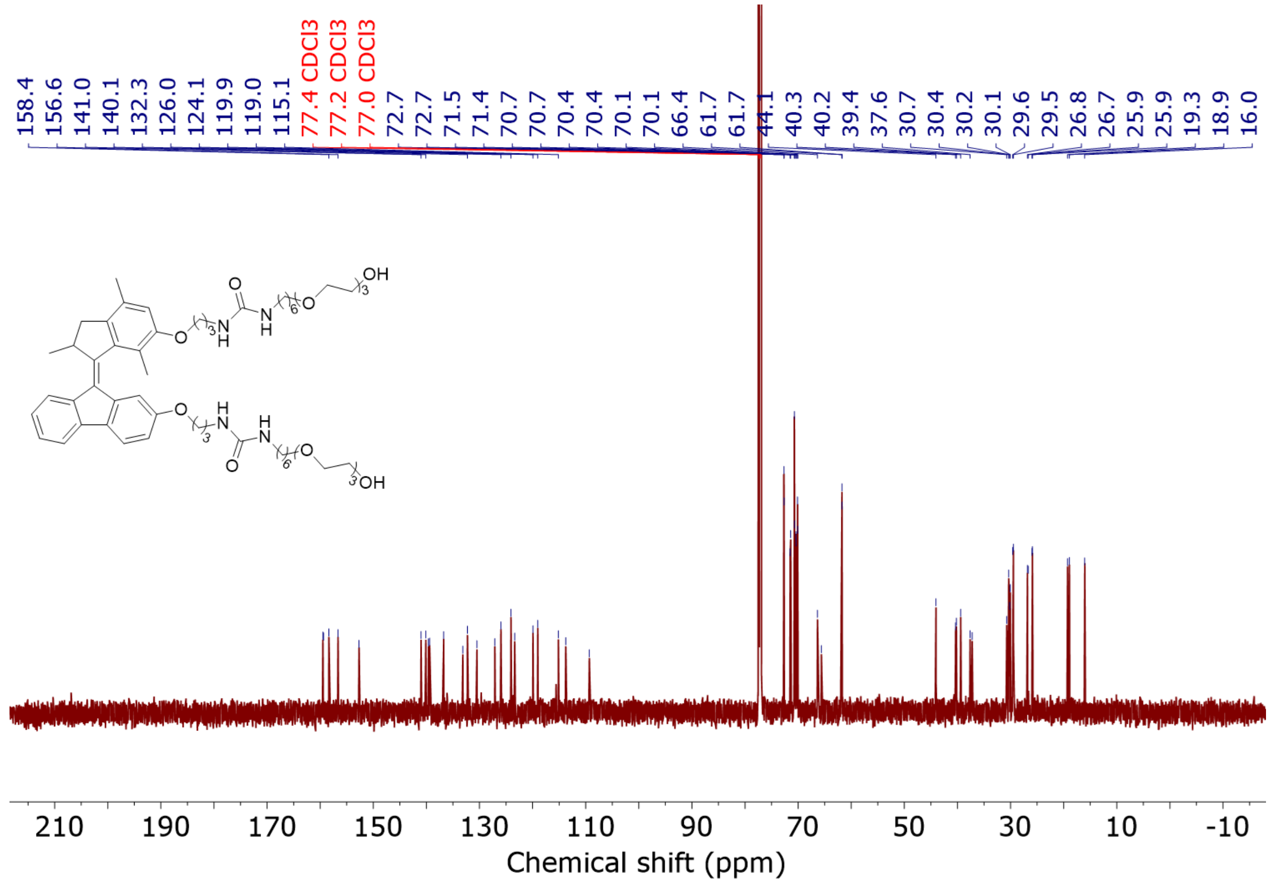


**Supplementary Figure 37.** $^{13}$C NMR spectrum of ***Z$_S$*-2MOEG3** (CDCl$_3$, 25 °C, 151 MHz).

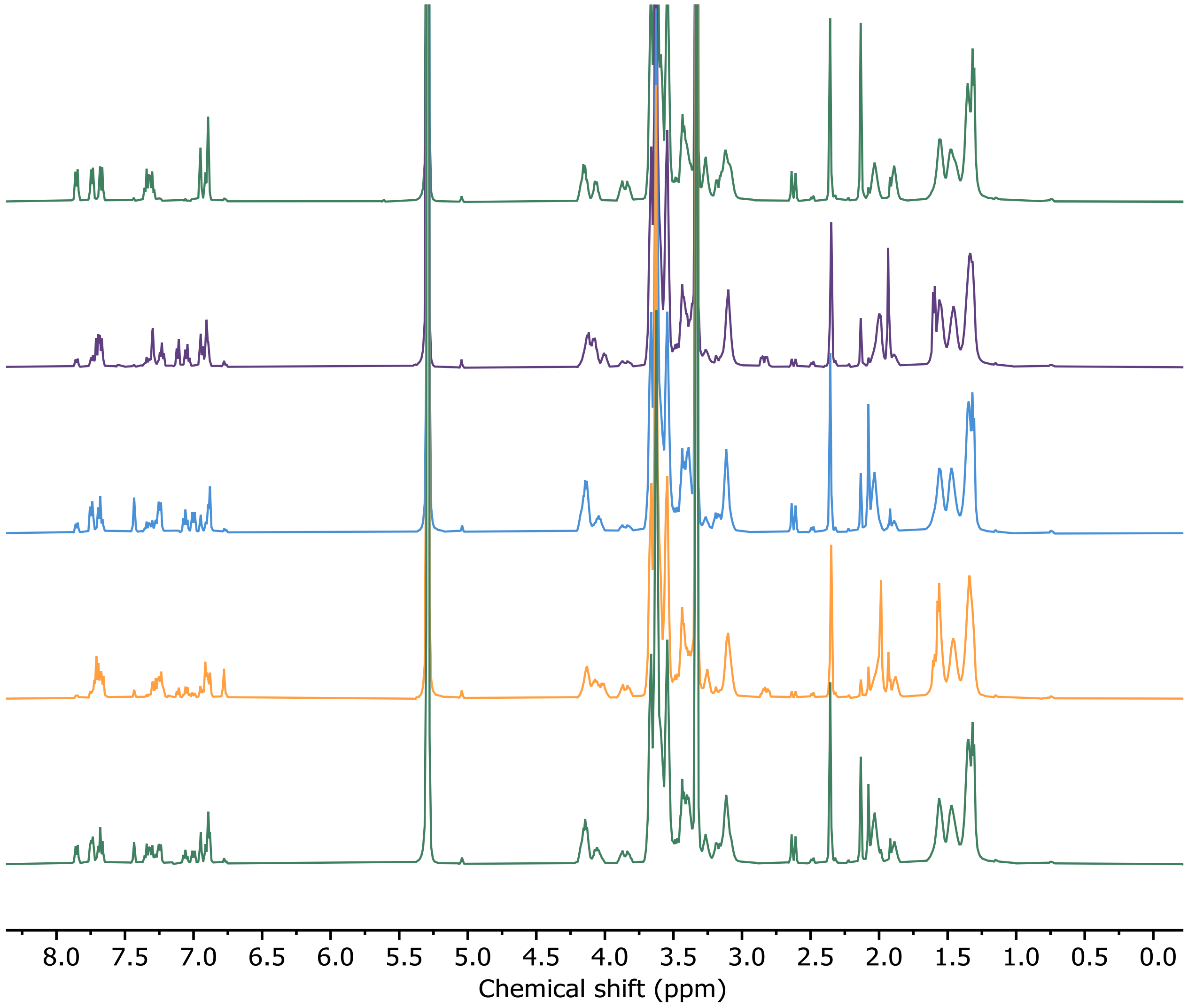


**Supplementary Figure 38.** $^{1}$H NMR spectra of the photoisomerization and THI process of ***Z$_S$*-2MOEG4**.

## 2. HRMS data

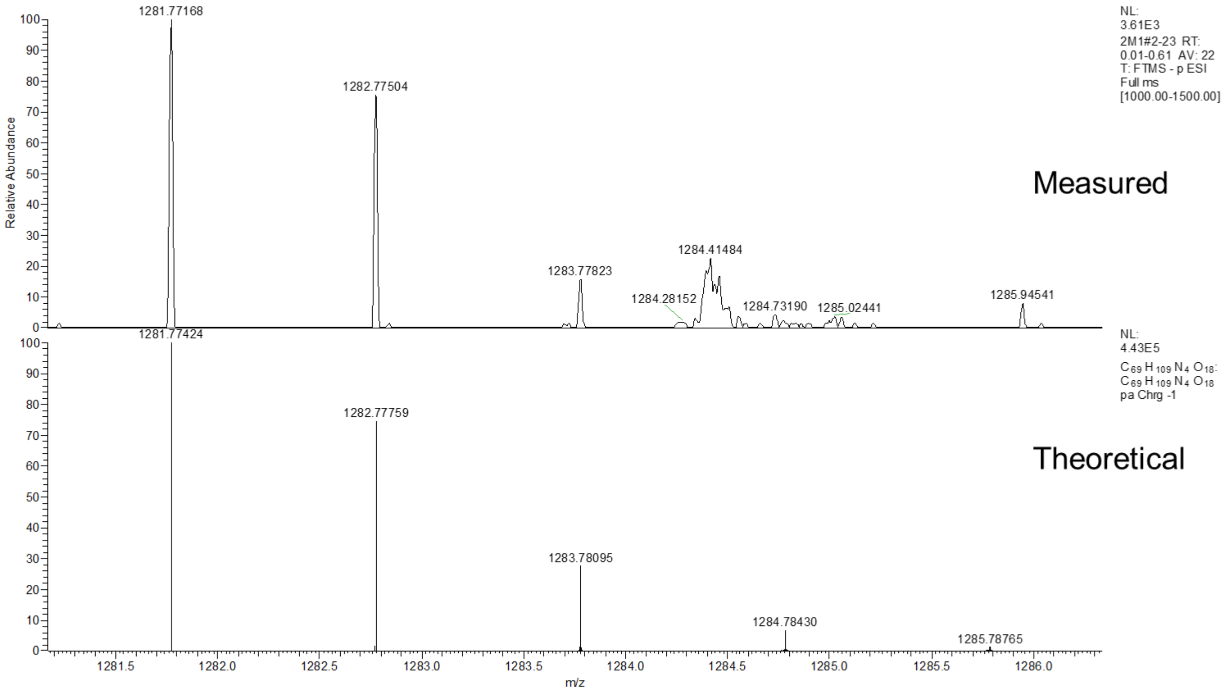


**Supplementary Figure 39.** HRMS (ESI+) data of ***$Z_S$*-2MOEG6** [M].

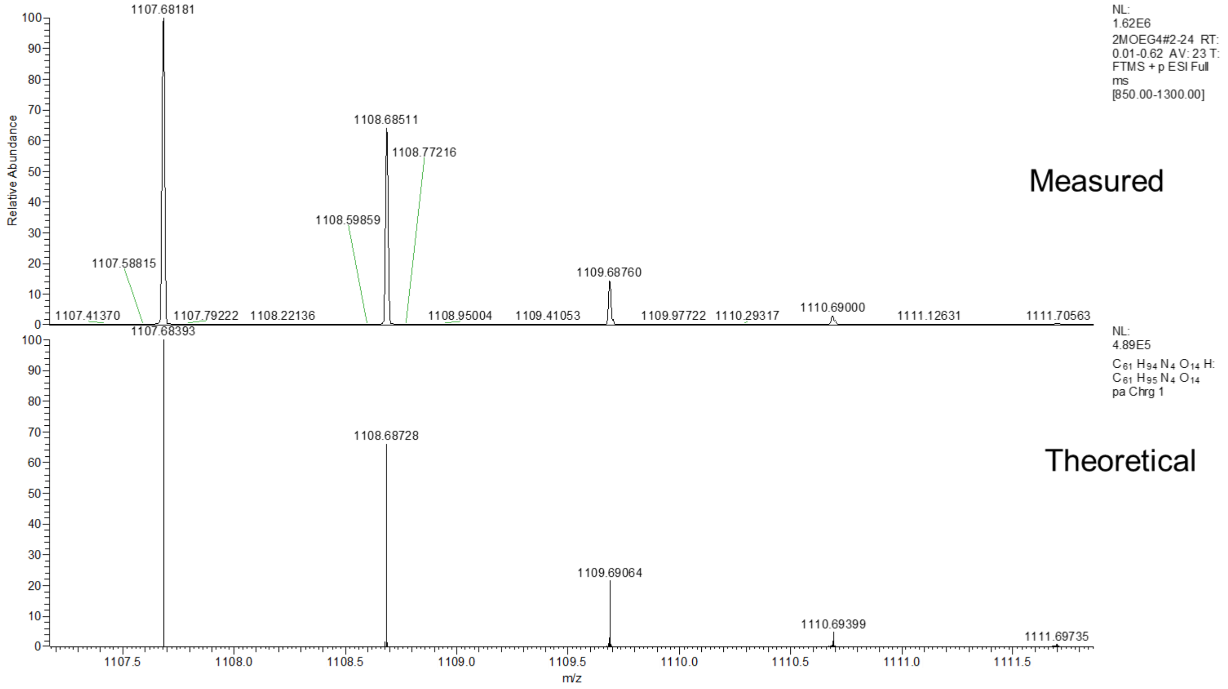


**Supplementary Figure 40.** HRMS (ESI+) data of ***$Z_S$*-2MOEG4** $[M+H]^+$.

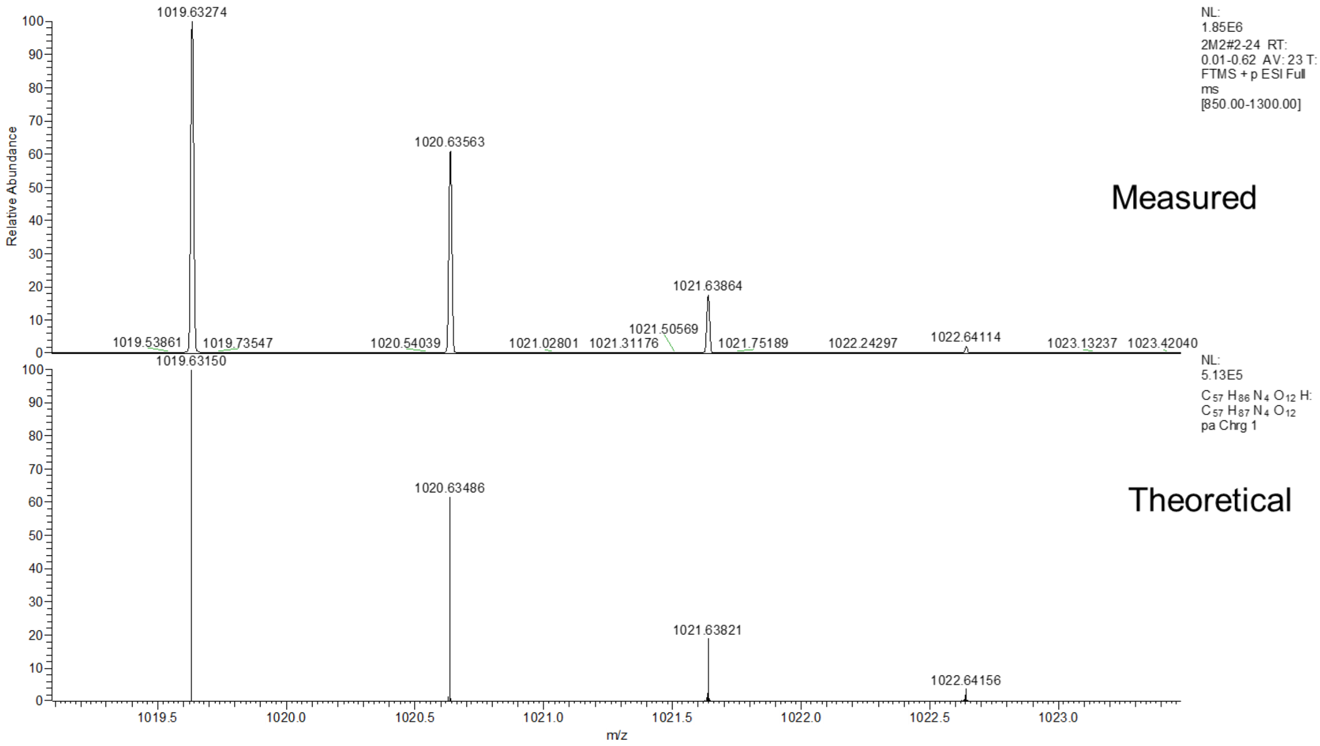


**Supplementary Figure 41.** HRMS (ESI+) data of ***Zs*-2MOEG3** [M+H]$^+$.

## Supplementary References


1. Hou, J. *et al.* Phototriggered Complex Motion by Programmable Construction of Light-Driven Molecular Motors in Liquid Crystal Networks. *J Am Chem Soc* **144**, 6851–6860 (2022).

2. Xu, F. *et al.* From Photoinduced Supramolecular Polymerization to Responsive Organogels. *J Am Chem Soc* **143**, 5990–5997 (2021).

3. van Ewijk, C. *et al.* Light-Triggered Disassembly of Molecular Motor-based Supramolecular Polymers Revealed by High-Speed AFM. *Angewandte Chemie International Edition* **63**, e202319387 (2024).

4. van Ewijk, C. *et al.* Light-Triggered Disassembly of Molecular Motor-based Supramolecular Polymers Revealed by High-Speed AFM. *Angewandte Chemie International Edition* **63**, e202319387 (2024).

5. Grimme, S., Ehrlich, S. & Goerigk, L. Effect of the damping function in dispersion corrected density functional theory. *J Comput Chem* **32**, 1456–1465 (2011).

6. Weigend, F. & Ahlrichs, R. Balanced basis sets of split valence, triple zeta valence and quadruple zeta valence quality for H to Rn: Design and assessment of accuracy. *Physical Chemistry Chemical Physics* **7**, 3297–3305 (2005).

7. Lu, T. & Chen, F. Multiwfn: A multifunctional wavefunction analyzer. *J Comput Chem* **33**, 580–592 (2012).
