# Supplementary material for: Molecular motor-driven reversible liquid-liquid phase separation of supramolecular assemblies
Source: Nat Commun. 2025 Nov 14;16:10017. doi: 10.1038/s41467-025-64993-9 (PMC12618938; doi:10.1038/s41467-025-64993-9)
Supplement: Supplementary file 2 — Description of Additional Supplementary Files [file 41467_2025_64993_MOESM2_ESM.pdf]

Description of Additional Supplementary Files

Supplementary Movie 1: Confocal laser scanning microscopy (CLSM) movie showing the dissolution of ZS-2MOEG4 droplets upon cooling from 60 °C to 25 °C. The movie is displayed at ×4 real-time speed.

Supplementary Movie 2: CLSM movie showing the formation of ZS-2MOEG4 droplets upon heating from 25 °C to 60 °C. The movie is displayed at ×8 real-time speed.

Supplementary Movie 3: CLSM movie showing the movement of ZS-2MOEG4 droplets at room temperature. The movie is displayed at ×8 real-time speed.

Supplementary Data 1: Atomic coordinates of the optimized molecular structures.
